# Using the canary genome to decipher the evolution of hormone-sensitive gene regulation in seasonal singing birds

**DOI:** 10.1186/s13059-014-0578-9

**Published:** 2015-01-29

**Authors:** Carolina Frankl-Vilches, Heiner Kuhl, Martin Werber, Sven Klages, Martin Kerick, Antje Bakker, Edivaldo HC de Oliveira, Christina Reusch, Floriana Capuano, Jakob Vowinckel, Stefan Leitner, Markus Ralser, Bernd Timmermann, Manfred Gahr

**Affiliations:** Department of Behavioral Neurobiology, Max Planck Institute for Ornithology, 82319 Seewiesen, Germany; Max Planck Institute for Molecular Genetics, Sequencing Core Facility, 14195 Berlin, Germany; Laboratório de Cultura de Tecidos e Citogenética, SAMAM, Instituto Evandro Chagas, Ananindeua, Pará, and Faculdade de Ciências Naturais (ICEN), Universidade Federal do Pará, Belém, 66075-110 Brazil; Department of Biochemistry and Cambridge Systems Biology Centre, 80 Tennis Court Road, Cambridge, CB2 1GA UK; Division of Physiology and Metabolism, MRC National Institute for Medical Research, the Ridgeway, Mill Hill, London, NW7 1AA UK

## Abstract

**Background:**

While the song of all songbirds is controlled by the same neural circuit, the hormone dependence of singing behavior varies greatly between species. For this reason, songbirds are ideal organisms to study ultimate and proximate mechanisms of hormone-dependent behavior and neuronal plasticity.

**Results:**

We present the high quality assembly and annotation of a female 1.2-Gbp canary genome. Whole genome alignments between the canary and 13 genomes throughout the bird taxa show a much-conserved synteny, whereas at the single-base resolution there are considerable species differences. These differences impact small sequence motifs like transcription factor binding sites such as estrogen response elements and androgen response elements. To relate these species-specific response elements to the hormone-sensitivity of the canary singing behavior, we identify seasonal testosterone-sensitive transcriptomes of major song-related brain regions, HVC and RA, and find the seasonal gene networks related to neuronal differentiation only in the HVC. Testosterone-sensitive up-regulated gene networks of HVC of singing males concerned neuronal differentiation. Among the testosterone-regulated genes of canary HVC, 20% lack estrogen response elements and 4 to 8% lack androgen response elements in orthologous promoters in the zebra finch.

**Conclusions:**

The canary genome sequence and complementary expression analysis reveal intra-regional evolutionary changes in a multi-regional neural circuit controlling seasonal singing behavior and identify gene evolution related to the hormone-sensitivity of this seasonal singing behavior. Such genes that are testosterone- and estrogen-sensitive specifically in the canary and that are involved in rewiring of neurons might be crucial for seasonal re-differentiation of HVC underlying seasonal song patterning.

**Electronic supplementary material:**

The online version of this article (doi:10.1186/s13059-014-0578-9) contains supplementary material, which is available to authorized users.

## Background

Seasonal behaviour, in particular the singing of birds, has fascinated human societies since ancient times (Aristotle in his *Historia Animalium*, approximately 350 BC) [1]. Songbirds are a large taxonomic group with a large degree of species variation to the extent that song-related behaviour depends on gonadal hormones, especially testosterone and estrogens [2-11]. In songbird species of the temperate zones, comprising about 2,000 species, singing is a seasonal sexual behaviour. Males of these species sing vigorously only or predominantly during the breeding season, which is determined by a long-day photoperiod. Further, in species with males that sing even outside of the breeding period, the songs of the breeding season have different temporal patterns or particular song types are uttered only in the breeding period (for example, [12,13]). Singing intensity, breeding-related song types and song pattern are dependent on the gonadal steroid hormone testosterone, which is produced in high levels during the breeding period. In temperate zone species, this testosterone surge follows a photoperiodic growth of the testicles (reviewed in [14]).

**Figure 1:**
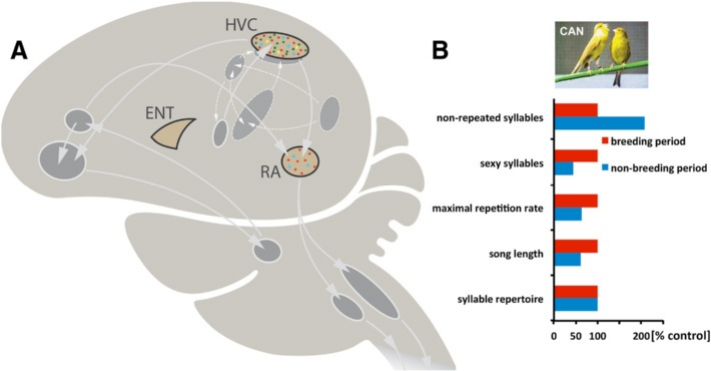
**Schematic of the song control system of songbirds and seasonal features of the canary song. (A)** HVC and RA are essential nuclei of the descending motor pathway of the song system that controls song production. All interconnected areas are parts of loops that feed back to the song motor pathway. The androgen receptors (red dots) are expressed in both the HVC and RA of canaries, while only HVC contains estrogen receptors (green dots) among song areas. Both areas express the 5α-reductase gene (blue squares) but only HVC expresses aromatase (yellow squares). The entopallium (ENT; the bird visual cortex) expresses neither androgen or estrogen receptors nor androgen- or estrogen-producing enzymes and was used as a contrasting brain area [20-23] (this study). **(B)** Song features such as song length, the number of non-repeated syllables, the number of sexy syllables and the maximal repetition rate of syllables change seasonally while the syllable repertoire of canaries does not change between their breeding and non-breeding season. The abundance of song segments with high repetition rates is a sexually attractive song pattern in canaries. Plotted are the percent differences between the breeding males (control) producing high testosterone levels and the non-breeding males producing low testosterone levels (redrawn after [16]).

The prototypical songbird species for seasonal singing is the canary (*Serinus canaria*) [12,15,16], a Carduelid songbird that originates from the Canary islands and was domesticated in the last centuries for various purposes, including being bred to win song contests [17]. The song of male canaries is highly stereotyped and includes syllables repeated with high frequency in the spring breeding season (long-day) while the songs uttered during the autumnal non-breeding period (short-day) are variable and contain few high-speed segments (Figure 1B). In wild and domesticated canaries, testis size and testosterone production are seasonally modulated, with high levels restricted to the breeding season, during which males sing intensely [12,15,16], a correlation that is confirmed experimentally by castration and testosterone treatment [3]. Estrogens, brain-derived metabolites of testosterone, are known to be specifically involved in the production of the canary song segments uttered with high repetition rates [18], which represent a key feature of the sexual attractiveness of songs for female canaries [19].

The singing behaviour of all songbirds is organized by the activity of the song control system (Figure 1A), which is similar among songbird species [24,25]. In the canary, in correlation with the seasonal testosterone-dependent plasticity of the singing behaviour, the song system undergoes large-scale seasonal neuronal and neural plasticity [26-29], including angiogenesis and neurogenesis [30,31]. Similar to other neural circuits that control sexual behaviours [32], the song system expresses androgen receptors (ARs) and estrogen receptors (ERα) [20,21,33]. These transcription factors are activated, respectively, by binding of the androgen testosterone and estrogens [34]. In the canary, ARs are expressed in most parts of the song system, while ERα expression is restricted to one song region, the HVC (Figure 1A) [20,35]. Thus, testosterone and its brain-derived estrogenic metabolites control singing behaviour via direct action in song control neurons. Although canaries are quite suitable to study seasonal behavioural and neuronal plasticity, the broad use of the canary model in molecular neuroscience has been limited so far by the lack of a high-quality reference genome sequence. The essential prerequisite for such analysis is the detailed knowledge of the canary genome sequence. The genomic adaptations of seasonal behaviour and gene expression are not well understood, neither in songbirds [36,37] nor in other vertebrates.

In songbirds, the evolutionary lineage and molecular genetics which resulted in the hormone responsiveness of singing behaviour are only barely understood. Meanwhile, the only bird genomes described in detail are those of closely related Galliformes/Anseriformes (the chicken [38], turkey [39] and duck [40]), as well as that of the zebra finch [41], the Tibetan ground tit [42] and the carrion crow [43]. Due to the large evolutionary distance between galliform/anseriform birds and songbirds [44], a comparison between these genomes is not informative with respect to the structures present in the canary brain and genome that are responsible for hormone-dependent plasticity of the song system. Further, the genomes of the zebra finch [41], the Tibetan ground tit [42] and the crow [43] are not helpful for discovering adaptations to testosterone-sensitive singing since their song pattern is either not hormone-sensitive (zebra finch [2,7,8,11]) or has not been investigated (Tibetan ground tit; carrion crow). We have generated a draft genome sequence for the canary in order to use it as a basis for transcriptional and proteomic analysis. We deeply sequenced and assembled the canary genome by integrating short- and long-read second-generation sequencing technologies. In contrast to previous sequencing of songbird genomes [41-43], we analysed the genome derived from a female canary because female birds are the heterogametic (Z/W) sex.

On the basis of this canary genome draft we analysed two major steroid hormone-sensitive song-control brain regions (HVC and RA) and one non-steroid hormone-sensitive, non-vocal but visual brain area (the entopallium (ENT)) via microarrays, RNA sequencing (RNA-seq) and targeted mass spectrometry (MS)-based proteomics (SWATH-MS). Subsequent to this global analysis we focused on gene networks (biological processes) that fulfil three criteria: being enriched (or under-represented) in the singing-related expression profiles of HVC and RA, being seasonal, and being testosterone-sensitive. In particular, we examined whether the genes of these gene networks contained elements (nucleotide sequences) that make them potentially more hormone-sensitive compared with canary genes not related to singing. To verify that the observed abundance of hormone-sensitive regulatory elements among such genes is canary-specific, we analysed whether these elements are absent in the structure of the orthologous genes of the zebra finch, since hormone-sensitive song pattern did not evolve in this species [2,7,8,11].

## Results

### The canary genome sequence

#### Whole genome shotgun sequencing, genome assembly, and scaffolding

**Figure 2:**
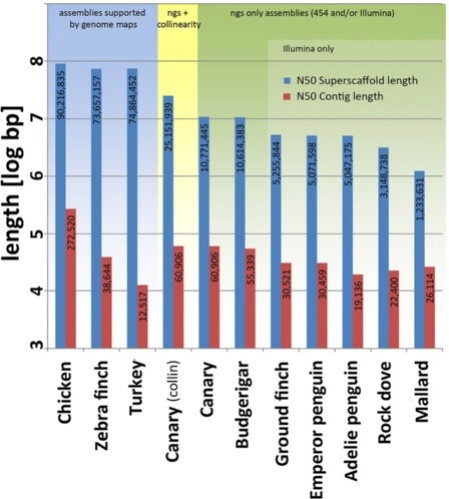
**Collinearity increases the superscaffold sizes of the canary genome significantly (yellow field) so that the assembly quality approaches that of the chicken, turkey and zebra finch, all based on additional sequencing information such as genetic linkage maps (blue field).** Note that the values are given as log bp. Without collinearity, the superscaffold length of the canary genome is among the highest among genomes sequenced with next generation technologies (ngs) only (green field). Note that details of the assembly procedures for the budgerigar, ground finch, emperor penguin, Adelie penguin, and rock dove have not been published. The N50 contig length is in red, and the N50 superscaffold length is in blue. For scientific species names and sources of genome assemblies see Materials and methods section M5.

Whole genome shotgun sequencing was carried out to generate high sequencing coverage (40×) of the canary genome based on short reads (2 × 100 bp or 2 × 115 bp), and lower coverage of the genome (5×) based on long reads (400 bp or 750 bp), and then assembled by CeleraAssembler6.1 (Additional file 1; Materials and methods sections M2 and M3). The canary genome assembly quality is reflected in its contig N50 length (60.9 kbp) and scaffold N50 length (10.77 Mbp) (Figure 2, Table 1). When comparing the assembly statistics of publicly available bird genomes (Figure 2; Additional file 2), many of the draft genomes which were sequenced/assembled by the Illumina technology alone (for example, ground finch, emperor penguin, Adélie penguin, rock dove) have about 50% lower values for contig N50 length and scaffold N50 length compared with projects in which Illumina and Roche/454 technologies were combined (canary and budgerigar). This underlines the benefit of long reads for gap closure or resolving difficult regions of the genome. However, recent improvements in short read genome assembly tools such as AllPath LG [31], increasing read length of the Illumina sequencing technology and optimized protocols for long insert sequencing library construction have enabled the assembly of bird genomes with higher contig N50 length from Illumina reads alone [42,43].

**Table 1:** **The quality of the canary genome assembly**

Contig number	62,459
N50 contig length	60,906
Contig length average	16,438
Largest contig	575,284
Degen contigs total length	104,692,948
Contigs total length	1,026,679,711
Superscaffold number	3,665
N50 superscaffold length	25,151,939
Superscaffold length average	286,906
Largest superscaffold	88,717,954
Superscaffolds total length	1,051,512,088

The assembly consists of 62,459 contigs with an N50 length of 60.91 kbp that add up to a consensus length of 1.027 Gbp. The contigs were ordered to 3,899 scaffolds with an N50 length of 10.77 Mbp. These could be combined to 3,665 superscaffolds by adding collinearity information from the zebra finch. The superscaffolds span a length of 1.051 Gbp and exhibit an N50 length of 25.15 Mbp. Because approximately 81% of the reads were assembled in the contigs, the complete genome of the canary should be approximately 1.2 Gbp in size. Data are given as bp.

Nevertheless, most of the assemblies mentioned above (Figure 2) are far from assembling scaffolds that are close to covering complete chromosomes. Due to the additional scaffolding via SSPACE, using the long-range mate pair data (Materials and methods section M4) and collinearity (Materials and methods sections M5 and M6) to the zebra finch chromosomes, the canary sequence was found to be among the highest quality genome assemblies of birds that were produced by second-generation technologies alone (Figure 2, Table 1). In particular, collinearity as a bioinformatics tool as described and evaluated in Materials and methods section M6 increased the scaffold (a set of contigs ordered by paired-end sequencing information) - now called a superscaffold (a set of scaffolds ordered by additional information on location/orientation) - N50 length of the canary by 2.5 times to 25.15 Mbp, although genetic linkage maps or other tools to improve long range continuity of the assembly were not available for the canary (Figure 2; Materials and methods section M6).

**Figure 3:**
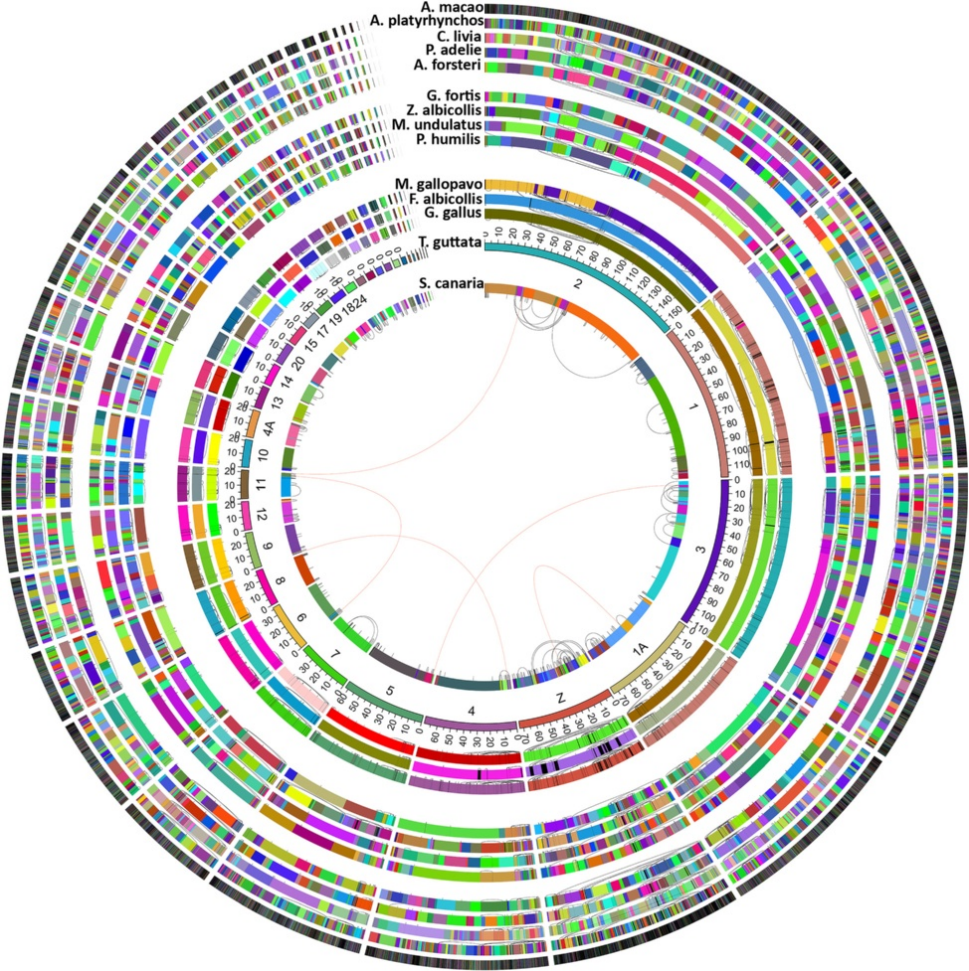
**Whole genome alignments of the canary genome and 13 other publicly available bird genome assemblies using the zebra finch genome as a reference underline the high long-range continuity of the canary genome assembly and highly conserved collinearity and synteny of genomes throughout the bird taxa.** Scaffold colours were chosen in a random fashion to visualize the assembly N50 length of the top level sequences (chromosomes, superscaffolds or scaffolds, depending on genome project), resulting in highly heterogeneous coloured plots for low quality genome assemblies (outside rings) and homogeneous coloured plots for high quality genome assemblies (inside rings). Black arcs depict putative intra-chromosomal rearrangements of the genome assemblies compared with zebra finch, many of which are found in different bird taxa and thus likely trace back to zebra finch-specific rearrangements or mis-assemblies in the zebra finch assembly. For the canary genome we also show five putative inter-chromosomal rearrangements (red arcs). For scientific species names and sources of genome assemblies see Materials and methods section M5.

Whole genome alignments of the canary genome and 13 other publicly available bird genome assemblies using the zebra finch genome as a reference underscore the high long-range continuity of the canary genome assembly and highly conserved collinearity and synteny of genomes throughout the bird taxa (Figure 3). We found 119, 114 and 107 putative intra-chromosomal rearrangements of synteny blocks that were supported by canary, ground finch [45] or white-throated sparrow [46], respectively, and at least one other species. Interestingly, 83 such rearrangements were present in two of the above passerine species and in at least one of the non-passerine species. Since passerines are a relatively young clade [47], we conclude that a high proportion of the rearrangements between passerine species may represent zebra finch specific rearrangements or more likely problems in the zebra finch assembly.

We also assigned several smaller scaffolds to the W chromosome (Table 2). These scaffolds showed no alignments with the zebra finch assembly because it is based on the male genome, which does not include the W chromosome [41]. In addition to the nuclear genome, the 16,809-kbp mitochondrial genome of the canary was assembled. These sequences have been submitted to the European Nucleotide Archive (ENA; accession number GCA_000534875 [48].

**Table 2:** **The genes of the W chromosome**

**Gene_ID canary**	**Symbols**	**Description (automated assignment)**	**Scaffold ID**	**Zebra finch paralogs**	**Medium ground finch paralogs**	**Canary paralogs**	**Chicken paralogs**
SCA_g3451(mRNA)	NIPBL	Nipped-B-like protein	SCA1_u120	1 (chrZ)	2 (no asgmts)	2 (putative Z/W)	2 (chrZ/chrW)
SCA_g3507(mRNA)	F1P4V8_CHICK	Valosin-containing protein [Source:RefSeq peptide;Acc:NP_001038129]	SCA1_u125	1 (chrZ)	2 (no asgmts)	2 (putative Z/W)	2 (chrZ/chrW)
SCA_g3994(mRNA)	-	Uncharacterized protein [Source:UniProtKB/TrEMBL;Acc:G1MSQ9]	SCA1_u139	1 (chrZ)	2 (no asgmts)	2 (putative Z/W)	2 (chrZ/chrW)
SCA_g4302(mRNA	SMAD2	SMAD family member 2 [Source:HGNC Symbol;Acc:6768]	SCAI_uI41	1 (chrZ)	2 (no asgmts)	2 (putative Z/W)	2 (chrZ/chrW)
SCA_g4352(mRNA)	RASA1	RAS p21 protein activator 1 [Source:HGNC Symbol;Acc:9871]	SCAI_u146	1 (chrZ)	2 (no asgmts)	2 (putative Z/W)	2 (chrZ/chrW)
SCA_g4788(ab initio)		PREDICTED: zinc finger SWIM domain-containing protein 6 [Gallus gallus]	SCA1 u157	No hits!	No hits!	1 (putative W)	2 (chrZ/chrW)
SCA_g5540(homology)	C18orf25	Uncharacterized protein C18orf25	SCA1u17O	1 (chrZ)	2 (no asgmts)	2 (putative Z/W)	2 (chrZ/chrW, chrUN)
SCA_g5588(mRNA)	UBAP2	Ubiquitin associated protein 2 [Source:HGNC Symbol;Acc:14185]	SCAI_u177	1 (chrZ)	2 (no asgmts)	2 (putative Z/W)	2 (chrZ/chrW)
SCA_g6069(homology)	KCMF1	E3 ubiquitin-protein ligase KCMF1 (EC 6.3.2.-)	SCAI_u186	1 (chrZ)	2 (no asgmts)	2 (putative Z/W)	2 (chrZ/chrW)
SCA_g8416(mRNA)	SPINZ_CHICK	Spindlin-Z [Source:UniProtKB/Swiss-Prot;Acc:Q90WG1]	SCAI_u248	1 (chrZ)	2 (no asgmts)	2 (putative Z/W)	2 (chrZ/chrW)
SCA_g8529(homology)	F1NGA2_CHICK	ATP synthase, H+ transporting, mitochondrial F1 complex, alpha subunit [Source:RefSeq peptide;Acc:NP_989617]	SCAI_u252	1 (chrZ)	2 (no asgmts)	2 (putative Z/W)	2 (chrZ/chrW)
SCA_g8946(mRNA)	TMED7 CGI-109	Transmembrane emp24 domain-containing protein 7	SCAI_i272	1 (chrZ)	2 (no asgmts)	2 (putative Z/W)	1 (chrZ)
SCA_g5568(mRNA)	SMAD4	SMAD family member 4	SCAI_u174	1 (chrUN)	2 (no asgmts)	2 (putative Z/W)	2 (chrUN/chr25)
SCA_g5589(mRNA)	-	Predicted: proto-oncogene c-Rel-like [Meleagris gallopavo]	SCA1_u177	Many	Many	Many	Many, best hit W
SCA_g5590(homology)	-	Predicted: hypothetical protein [Taeniopygia guttata]	SCAI_u177	Many	Many	Many	Many
SCA_g8399(homology)	D7F039_TAEGU	Fem1c-Z [Source:RefSeq peptide;Acc:NP_001177271]	SCAI_u243	1 (chrZ)	2 (no asgmts)	1 (putative W)	1 (chrZ)
SCA_g8945(ab initio)	-	Fem1c-Z [Taeniopygia guttata]	SCA1u272	1 (chrZ)	2 (no asgmts)	1 (putative W)	1 (chrZ)
SCA_g15017(homology)	-	Uncharacterized protein	SCAI_u533	1 (chrUN)	1 (no asgmts)	1 (putative W)	1 (chrW)

The assembled canary W chromosome superscaffolds add up to 1.69 Mbp and thus far contain 12 genes (chicken has 21 genes and 5 RNA genes [49], of which three genes (TMED7, F1P4V8_Chick, and FEM1C_Z) are Z linked in the chicken genome). The assignment of these genes to the W chromosome is supported by the findings that (i) most of these genes have only one paralog in the zebra finch genome assembly, which is lacking the W chromosome, and (ii) one ortholog and one paralog are found on chrZ/chrW of the canary, the chicken and the medium ground finch. In these species, the W chromosome was included in genomic sequencing, although no scaffolds were assigned to chromosomes in the case of the medium ground finch. No asgmts = no assignments.

Our decision to sequence a female canary to gain information on chromosome W has drawbacks regarding the quality of the assembled Z chromosome sequence, as only half of the average sequencing coverage is available for assembly of the heterosomes. As expected, N50 contig and N50 scaffold values of the assembled Z sequences are lower compared with the autosomal part of the canary genome assembly (Additional file 3). However, the colinearity-based superscaffolding overcame this drawback and improved the canary Z scaffolds (N50 of superscaffolds; Additional file 3) to sizes that equal the zebra finch Z assembly. Thus, we suggest that high-quality bird genome assemblies are possible based on heterogamous females if appropriate bioinformatic tools are employed for genome assembly and in case of high coverage.

#### Repeat analysis and CpG island prediction

A total of 13.25% of the assembled contigs are masked by the RepeatModeler/RepeatMasker software. Superscaffold contig sequence (representing 90.75% of total contig length) is masked to a lower extent than degenerate contig sequence (9.4% and 51.9%, respectively). This reflects that the short degenerate contigs - those contigs from the Celera assembler output that cannot be reliably placed in scaffolds - contain hard to assemble regions of the genome. About 14.9% of the masked repeat sequence in the superscaffold contigs was newly identified by the RepeatModeler software as repeat masking with the Repeatmasker libraries (known repeats from other species) masked only about 8% of the superscaffold contigs.

Of transposable elements, LINES (long interspersed elements) account for 3.65% and LTR (long terminal repeat elements) for 4.7% of the assembly. Unclassified repeats make up 3.14% of the sequence. Small RNA, satellites, simple repeats and low complexity sequence comprise only a minor part of the masked sequence (0.03%, 0.2%, 0.74%, and 0.73%, respectively). CpG island prediction results in 35,007 islands with a length of 200 bp or more. These make up 1.12% of the genome and 8,572 CpG islands are located upstream of annotated genes (1,000 bp window).

#### The canary karyotype

Despite the high quality assembly, with chromosome-sized superscaffolds, there is obviously a mismatch between real chromosome numbers found in the karyotype of canaries (N = 40; Additional file 4) and the number of assembled chromosomal groups (N = 35). This difference is due to the large number of micro-chromosomes in bird species. In particular, the chicken genome comprises 33, the zebra finch 35 [41] and the canary 35 assembled chromosomes. In contrast to the chicken karyotype, which consists of a haploid set of 39 chromosomes [50-53], we found 40 chromosomes in canaries (Additional file 4), concordant with Ohno *et al*. [54]. Despite these mismatches, comparisons of chromosome painting between chicken and zebra finch indicate that the genomic location of chicken genes widely predicts the chromosomal location of the zebra finch orthologs [55], and presumably the canary orthologs.

#### Genome annotation and canary genome browser

The combined data from the assembled transcripts and protein homology revealed 16,294 protein-coding gene models, of which 12,246 (75%) resulted from transcriptome assemblies (Materials and methods section M10) and 4,048 (25%) from mapping protein sequences to the canary genome via SPALN (Materials and methods section M8). Additionally, 2,524 non-redundant gene models predicted using AUGUSTUS software were included in the reference gene set, resulting in 18,818 protein-coding genes and 3,882 potentially non-coding RNAs, excluding the mitochondrial genes. For the zebra finch, 17,475 genes have been proposed [41]. In total, our gene models cover 506.3 Mbp (44.75%) of the canary genome assembly. In detail, 65.2 Mbp (5.76%) of the assembly is covered by exons, of which 26.7 Mbp (2.36%) is coding sequence, 7.63 Mbp (0.67%) is 5′ UTR and 32.64 Mbp (2.89%) is 3′ UTR, while introns comprise 446.7 Mbp (39.47%) (Materials and methods section M9). To facilitate the analysis of the genome for public users, we set up a genome browser based on the UCSC-GB interface [56,57].

In order to analyse the testosterone and estrogen responsiveness of the singing-related genome, we next identified seasonal testosterone-sensitive transcriptomes of HVC and RA.

### Transcriptome and proteome related to hormone sensitivity of song control areas

Previous transcriptome studies of song control areas of different songbird species by means of microarrays showed mixed evidence for the enrichment of steroid hormone-sensitive gene networks [37,41,58-62]. A meta-analysis of these studies did not produce evidence of genomic adaptations related to hormone sensitivity [36]. In our study, the transcriptomes of HVC and RA (Materials and methods section M10) were analysed in relation to season and testosterone, and in relation to AR- and ERα-based hormone sensitivity of brain areas.

#### Seasonal testosterone-sensitive transcriptomes of the canary HVC and RA

First we compared the HVCs of long-day (LD) males, short-day (SD) males, and short-day males treated with testosterone (SD + T) using microarray procedures. LD males are reproductively active and produce highly stereotyped songs. SD + T males also sing such songs while normal SD males sing variable, so-called plastic or autumnal songs. SD males have degenerated testes and low-to-no testosterone production in contrast to LD males (Materials and methods section M1) [16].

**Figure 4:**
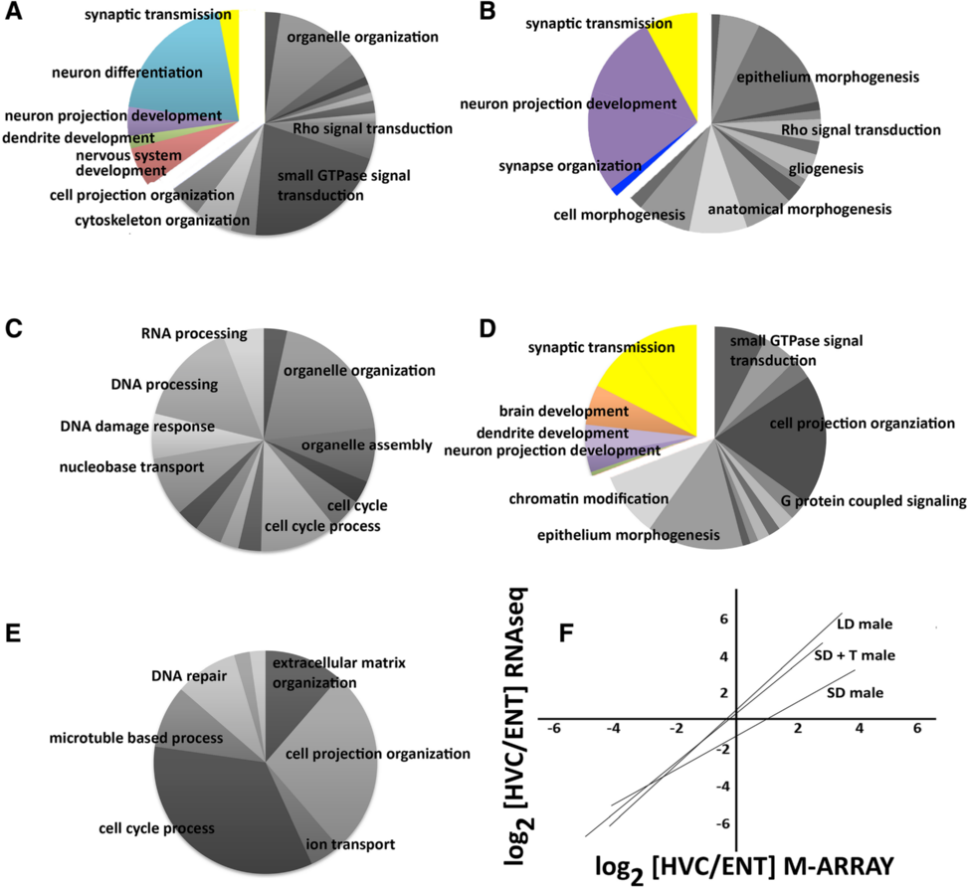
**Gene ontology of HVC transcriptomes (A-D) and RA transcriptomes (E) related to season (A,C,E), to testosterone (B), and to the area-specific presence/absence of androgen and estrogen receptors (D) (see** Materials and methods **sections** M10 **,** M11 **and** M15 **for details).** Biological processes typical of neuronal differentiation and synaptic transmission are depicted in colour; all others are depicted in grayscale. Due to space limitations, we could not include the names of all significant biological processes in the charts, but we list them in Additional file 5. **(A)** Of the seasonal biological processes of up-regulated genes of LD HVC versus SD HVC, 34.9% (sum of the coloured segments) relate to synaptic transmission and neuronal differentiation, which includes neurogenesis. **(B)** Testosterone-induced biological processes of up-regulated genes of HVC of SD + T males versus SD HVC; 36.8% of the biological processes are related to synaptic transmission and neuronal differentiation. **(C)** None of the seasonal biological processes of down-regulated genes of LD HVC versus SD HVC concern typical neuronal categories. **(D)** Area-specific biological processes of up-regulated genes of LD HVC versus LD ENT; 30.6% of these HVC-specific processes relate to synaptic transmission and neuronal differentiation. **(E)** Seasonal biological processes of up-regulated genes of LD RA versus SD RA concern general cell biological processes but not neuronal differentiation. **(F)** Differentially expressed HVC transcriptomes (genes that are significantly up- or down-regulated in HVC versus ENT) depend on elevated levels of testosterone. The ‘LD male’ curve shows the good agreement between the two techniques for assessing the differential expression of HVC genes (R^2^ = 0.85) of different groups of LD males, whereas the transcriptomes do not correlate well between LD and SD males (‘SD male’ curve with R^2^ = 0.46) but do so between LD and SD + T males (‘SD + T male’ curve with R^2^ = 0.79).

Gene Ontology (GO) analysis of the HVC transcriptomes using ClueGo [63] (Materials and methods section M15) showed that, overall, the significant biological processes for LD and SD + T males are rather similar. Of the biological processes of the specifically up-regulated HVC genes of LD males compared with SD males, 34.9% are related to neural and neuronal differentiation (including neurogenesis) and synaptic transmission (Figure 4A; Additional file 5). In the SD + T to SD comparison, the fraction of biological processes associated with these categories is similar (36.8%; Figure 4B; Additional file 5). There are, however, differences among these categories - for example, dendrite development and overall neuron differentiation are more represented in the LD HVC while neuron projection development and synapse organization are more abundant in the SD + T HVC. The most notable differences between LD and SD + T males concern the importance of organelle organization and the regulation of small GTPase-mediated signal transduction pathways in the HVC gene networks of LD males and epithelial morphogenesis (including angiogenesis) and gliogenesis and related networks in the HVC of SD + T males. Since gliogenesis and angiogenesis are induced in the HVC of female canaries following testosterone treatment [30,64] and are likely to occur seasonally, certain testosterone-induced gene networks may be transiently active, resulting in their absence in LD males, or LD males might be physiologically less synchronized than the SD + T males. A similar reasoning might explain the small differences in the neural categories for LD and SD + T males mentioned above. Most significantly, the genes that are more highly expressed in the SD HVC are not associated with typical neuronal processes but are mainly related to the cell cycle process, DNA repair, RNA processing and organelle organization (Figure 4C; Additional file 5). Furthermore and contrary to HVC, seasonal differences in the transcriptomes of RA do not suggest seasonality of neuronal differentiation and synaptic activity (Figure 4E; Additional file 5).

#### HVC and RA area-specific transcriptomes of long-day male canaries

Next we studied the HVC- and RA-specific transcriptomes of LD males by comparing these testosterone-sensitive brain areas with the ENT (Additional file 1). The ENT is a visual forebrain region that does not express AR or ERα and is not reported to undergo hormone-dependent or seasonal neuroplasticity [20]. Contrasting HVC and RA with the ENT is reasonable in order to detect ERα and testosterone, that is, AR-sensitive singing-related transcriptomes.

Detailed GO term analysis (ClueGo; Materials and methods section M15) of up-regulated area-specific HVC genes showed that 13.4% of the biological processes are related to neuronal differentiation and 17.2% to synaptic transmission (Figure 4D; Additional file 5). Area-specific non-neuronal biological processes were primarily related to cell signalling, adhesion and cell projection organization (Figure 4D). The analysis of down-regulated HVC genes resulted in few significant (*P* < 0.05) biological processes, mainly cellular protein metabolic processes, cell projection organization and cellular localization (not shown). The results for area-specific biological processes of RA were rather similar with a somewhat lower importance of neuronal differentiation (5.7% nervous system development, 14.6% synaptic transmission) compared with those of the HVC (Additional file 5).

As expected from previous work [20,22,65,66], the AR, ESR1 and CYP19A1 (coding for aromatase, which converts testosterone into estrogen) genes were highly expressed in the HVC compared with the ENT (Figure 1A). Further, we found *SRD5A2*, the gene coding for 5α-reductase, which converts testosterone into 5α-dihydrotestosterone, in HVC but not in ENT. In contrast to HVC, RA expressed AR and SRD5A2 genes but neither ESR1 nor aromatase.

#### Seasonal testosterone-sensitive HVC-specific gene networks

**Figure 5:**
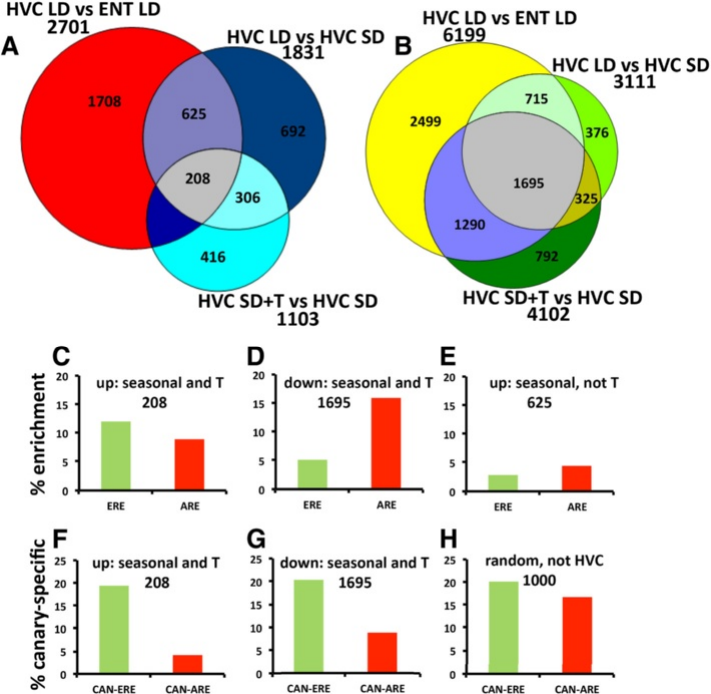
**Hormone sensitivity of seasonal transcriptomes. (A,B)** Venn diagrams of up-regulated transcriptomes **(A)** and down-regulated transcriptomes **(B)** related to season (HVC LD versus HVC SD), testosterone (HVC SD versus HVC SD + T), and area-specificity (HVC LD versus ENT LD) (Figure 4). **(C-E)** From the various resulting transcriptomes of these comparisons, we calculated the frequency of genes with an androgen response element (ARE) or estrogen response element (ERE) in their promoters. Numbers below the headers of **(C-G)** relate to the differential transcriptomes of **(A)** and **(B)**. Many more genes are down-regulated **(B)** compared with up-regulated **(A)** in each of the comparisons: 833 genes are up-regulated seasonally in HVC, among which 208 are testosterone-inducible; 2,410 are down-regulated seasonally in HVC, among which 1,695 are testosterone-inducible. Genes containing ERE are enriched particularly among the testosterone-sensitive seasonal gene pool **(C)** compared with seasonal but not testosterone-sensitive genes **(E)** or random (not shown) gene pools. Among the down-regulated transcriptomes, AREs are particularly enriched among the testosterone-sensitive seasonal genes **(D)**. The frequency of AREs and EREs in **(D,E)** is depicted as the percentage enrichment compared with ARE and ERE abundance in genes not expressed in HVC. **(F-H)** The frequency of canary-specific AREs (CAN-ARE) and of canary-specific EREs (CAN-ERE) among the various gene pools. Canary-specific means the AREs and EREs are absent in orthologous genes of the zebra finch genome. Percentage is based on all genes of a gene pool (here 208, 1,695 and 1,000; see Additional file 6 for gene lists). Note that AREs of testosterone-sensitive seasonally expressed genes **(F,G)** are conserved, that is, the frequency of CAN-AREs is much lower compared with genes not expressed in HVC **(H)**. This is not the case for CAN-EREs.

Due to the absence of seasonal neuronal categories for RA (Figure 4E), we did not analyse testosterone sensitive RA-specific gene networks as we describe in the following for HVC. The differentially expressed genes of the HVC of LD males do not correlate well with those of SD males (R^2^ = 0.46; Figure 4G) but do correlate after testosterone treatment of SD males (LD versus SD + T with R^2^ = 0.79; Figure 4F). The comparison between the seasonal HVC transcriptome, the testosterone-induced HVC transcriptome, and the area-specific HVC transcriptome (Figure 5) shows that 44% of the testosterone-sensitive categories are seasonal (Additional file 5) and that most of the testosterone seasonal categories are related to neuronal processes (see below). The main differences between seasonal and testosterone-induced HVC-specific processes are not directly related to neuronal differentiation but concern overall morphogenesis, including blood system development, intracellular organization, and signalling pathways (Figure 4A,B; Additional file 5).

**Figure 6:**
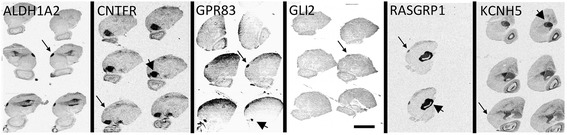
***In situ*** **hybridization for mRNA of genes differentially expressed in HVC, RA or entopallium.** Expression, either up- or down-regulated in HVC and RA compared with the ENT, was as expected from the RNA-seq for all six depicted genes. Next to the high expression in HVC (small arrows for ALDH1A2, CNTFR, GPR83, GLI2, RASGRP1) or RA (large arrow for GPR83) of LD canaries, the selected genes are expressed in other, distinct brain regions, such as the medial striatum (large arrow for RASGRP1), the arcopallium (large arrow for CNTFR) and the mesopallium (large arrow for ALDH1A2). KCNQ5 is down-regulated in the HVC of the depicted SD canary but year-round highly expressed in the ENT (large arrow). Abbreviations: ALDH1A2, aldehyde dehydrogenase 1 family, member A2; CNTFR, ciliary neurotrophic factor receptor; GLI2, GLI family zinc finger 2; GPR83, G protein-coupled receptor 83; KCNH5, potassium voltage-gated channel, subfamily H (eag-related), member 5; RASGRP1, RAS guanyl releasing protein 1 (calcium and DAG-regulated). Shown are photomicrographs of autoradiograms of sagittal sections of the canary brain. Labelled areas appear darkened. The scale bar equals 5 mm.

Among the seasonally and testosterone up-regulated genes, 208 are HVC-specific (Figure 5A; Additional file 6). Interestingly, 85% of the latter genes are related to neuron differentiation, axon, dendrite and synapse organization but not to neurogenesis (Additional file 6; Figure S2A in Additional file 7) For example, ROBO2 [67] and SLIT2 [68] are likely involved in neurite outgrowth or axonal pathfinding, which are morphogenetic reactions of HVC to long-day photoperiod and testosterone. The sodium leak channel UNC80 is important for neuronal excitability related to rhythmic behaviours [69], which is the foremost function of HVC. The functions of RASGRPs (Figure 6) in vertebrate neurons are unknown but might couple external stimuli to behaviour [70]. Further, the testosterone-sensitive seasonally up-regulated genes include 25 transcription factors (Additional file 6), among which PPARGC1 (peroxisome proliferator-activated receptor gamma, coactivator 1 alpha) is an ERα coactivator [71] and ZMIZ1 (zinc finger, MIZ-type containing 1) is an AR coactivator [72]. FOXP1, a human speech-related transcription factor, was previously reported to be highly expressed in HVC of zebra finches [73] and Bengalese finches [74], although its role in singing is unclear.

Contrary to the up-regulated genes, most of the 1,695 down-regulated genes (Figure 5B; Additional file 6) that are seasonal, testosterone-induced, and HVC-specific are not involved in typical neuronal processes but are involved in the cell cycle process, organelle organization and extracellular structure organization (Additional file 5; Figure S2B in Additional file 7).

In summary, since testosterone is high in SD + T and LD males, since HVC but not ENT is a direct target of AR- and ERα-mediated activity of testosterone, and since HVC is involved in song patterning but not ENT, we suggest that the 208 up-regulated HVC-specific, seasonal, testosterone-induced genes are crucial for neural and neuronal mechanisms of HVC underlying seasonal song patterning.

#### Area-specific expression of HVC and RA transcripts in LD canaries are confirmed by RNA-seq and in situ hybridization, and translate into differential expression of the encoded proteins

Because transcriptional profiling is sensitive to the presence of contaminant tissues, as well as changes in RNA content, RNA localization, cell size, and cell density, we performed a series of experiments to validate the altered expression of hormone-responsive genes between the HVC and the ENT (Additional file 1). First, the GO analysis (ClueGo) of the genes up- or down-regulated significantly in HVC compared with ENT confirmed the microarray-based results for HVC up- and down-regulated biological processes. Second, the area-specific transcriptomes of LD males resulting from microrarrays were compared with data obtained from RNA-seq analysis to exclude technical bias. The expression data obtained with both technologies (RNA-seq versus microarray of (HVC/ENT): R^2^ = 0.85 (Figure 3G)) were in agreement, demonstrating that both technologies identified tissue-specific changes in mRNA expression (Materials and methods sections M10 and M11). In particular, of the seasonal, testosterone-sensitive, and HVC-specific up- or down-regulated genes found with the microarray, we confirmed 72% with RNA-seq. Third, we demonstrated that genes differentially expressed in the HVC, RA or ENT using RNA-seq and microarrays are expressed in specific areas by *in situ* hybridization of brain sections (Figure 6; Materials and methods section M12). For genes found differentially expressed by both microarray analysis and RNA-seq, confirmation with *in situ* hybridisation was successful in all cases.

**Figure 7:**
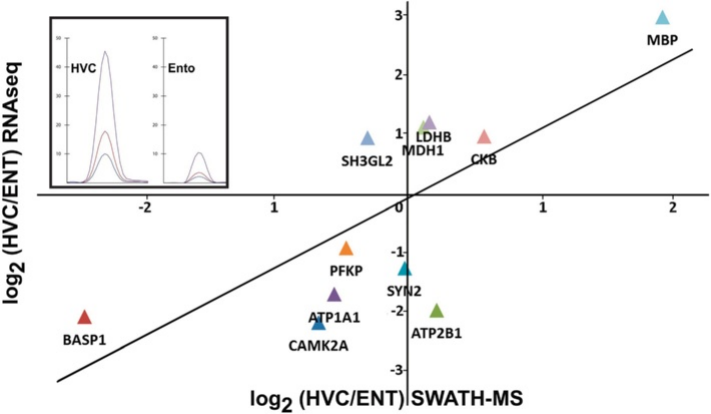
**RNA-seq-based HVC gene expression correlated with HVC protein SWATH-MS results.** The data were normalized to the expression levels of the ENT; positive values indicate higher expression in HVC. For 10 of the 13 investigated proteins, the correlation between protein and mRNA abundance is strong (r = 0.76). Nonetheless, we also found examples where the post-translational control of gene expression appears to be crucial: the endophilin SH3GL2 had a substantially higher protein expression level (as predicted from its RNA level), whereas levels of the calcium-transporting ATPase ATP2B1were lower than its mRNA expression would indicate. The insert shows an exemplary peptide chromatogram from SWATH-MS [77] for the peptide ASDPAAPPEEAK, which is specific for the protein MBP and is less expressed in the ENT compared with the HVC. Y-axis: intensity cps × 10^3^.

As changes in a transcriptome are, in many cases, not predictive of changes in the proteome due to different turnover rates, translational control and protein degradation [75], we studied the expression levels of identified proteins by the targeted proteomic technique SWATH-MS [76,77]. We quantified protein expression in both HVC and ENT of LD males and compared relative protein with transcript abundance (Figure 7; Materials and methods sections M13 and M14). Overall, there was a correlation of protein and RNA abundance with R^2^ values similar to what has been described in mammalian studies [75], indicating that, overall, large changes in the transcriptome were translated into changes in expression of the encoded proteins (Figure 7).

These results indicate that canary HVC microarray and RNA-seq expression levels are reliable predictors for singing-related protein abundance and that differential expression compared with the ENT is due to the area-specific distribution of mRNA.

Next, we studied the testosterone and estrogen sensitivity of the HVC transcriptome at the genomic level.

### Genomic adaptations related to the hormone sensitivity of the song system of canaries

#### Regulatory novelties of singing: estrogen and androgen response elements are enriched in genes that are testosterone-dependent and seasonally regulated in the HVC

A dominant mechanism of AR or ER transcriptional activity occurs via canonical DNA binding sites: the androgen response element (ARE) and the estrogen response element (ERE) [34]. Other seasonal transcriptional activity of ERα in the HVC, such as tethering to the transcription factors AP1 and SP1 [78,79], is unlikely because these genes are not seasonally expressed in HVC. We calculated the frequency of genes with *cis*-regulatory AREs and/or EREs among up- or down-regulated (seasonal and/or testosterone-induced) genes of the various HVC transcriptomes defined in Figure 5. For this we identified the promoters of such genes based on the RNA-seq-based assembly of the HVC transcriptome. Then, we compared the promoters of such genes from canaries with those from zebra finch orthologs. For these genomic comparisons, we only analysed the promoters of genes without sequence gaps in both the canary and zebra finch orthologs (Materials and methods section M16). To this end, we RNA-sequenced and assembled the transcriptome of the HVC of male zebra finches (Additional file 1; Materials and methods section M10).

First, in cases where genes contained AREs and/or EREs, the average number of promoters with AREs was three to four and of promoters with EREs was two to three. Second, we calculated the enrichment of AREs and EREs among the genes up-regulated (Figure 5A) or down-regulated (Figure 5B) specifically in the HVC either seasonally or by testosterone induction or both (see Additional file 6 for gene lists). Among genes up-regulated in both LD males and SD + T males, the frequency of genes with AREs and EREs was 9% and 12% higher, respectively (Figure 5C), compared with randomly selected genes (see Additional file 6 for gene list) assumed to be a genomic baseline. Such enrichment of genes with AREs or EREs was not seen in HVC up-regulated genes that are seasonal but not testosterone-induced (Figure 5E), and was not found in genes that are not seasonal (data not shown). For seasonal and testosterone-induced genes that were specifically down-regulated in HVC (Figure 5B), the result was a 16% increase of genes with AREs and a 5% increase of genes with EREs (Figure 5D). Thus, AR and ERα might directly control many of the HVC genes that are both seasonally and testosterone-dependently regulated. Third, we analysed the canary-specificity of ARE- and ERE-containing promoters. Among all HVC expressed genes, including the seasonal and testosterone-induced gene pools, 20% had EREs in canary promoters but not in the zebra finch orthologs (Figure 5F,G). In contrast to the EREs, the frequency of canary-specific AREs was 18% in gene pools not expressed in HVC (Figure 5H) but only 4% in up- and 8% in down-regulated seasonal testosterone-dependent transcriptomes (Figure 5F,G). Fourth, the genes with canary-specific EREs and AREs among these seasonally, testosterone up-regulated HVC genes were categorized exclusively as neuronal differentiation processes (Additional files 5 and 6).

**Figure 8:**
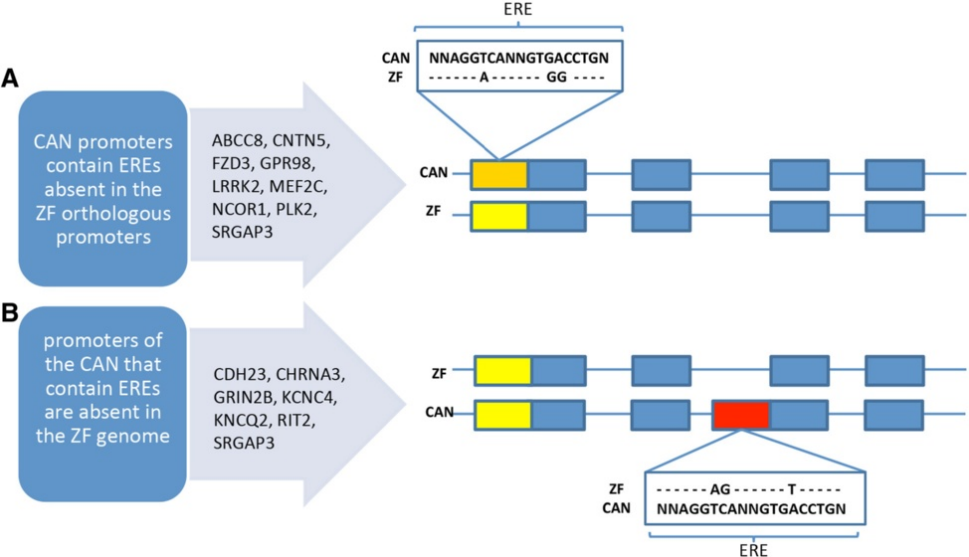
**Canary-specific estrogen response elements (see inserts) occur in the orthologous promoters of canary and zebra finch genes. (A)** Genes with orthologous promoters but that contain nucleotide sequences in zebra finch deviating from known ERE sequences (see insert in (A)). **(B)** Genes with EREs in the promoters of canaries for which we did not find orthologous promoters in zebra finch. In these cases, sequence analysis of 1 kb of the putative promoter region of the zebra finch did not identify EREs. The 15 genes analysed in detail were randomly selected from the list of genes with canary-specific EREs. Thus, of the about 550 seasonally up-regulated genes that contain canary-specific EREs, about one-half are classified as type ‘A’ and the other half as type ‘B’. Some genes such as SRGAP3 are in both categories. Orthologous promoters are in yellow, those containing EREs are in orange, and heterologous promoters are in red.

The reasons for this species difference in putative estrogen receptor binding sites were studied in detail for 15 genes randomly selected from the pool of genes containing EREs only in canaries (Figure 8). In about half of these genes, nucleotide differences in the orthologous promoter of the zebra finch are incompatible with the ERE canonical sequences (Figure 8A). These canary-specific EREs are likely to originate from point mutations since we did not find signs of transposable elements at ARE and ERE sites. In the other half of the analysed genes with EREs only in canaries (Figure 8B), we found EREs in additional promoters without orthologs in the zebra finch transcriptome.

## Discussion

### The canary genome sequence: protein-coding genes of the hormone-dependent, singing-related transcriptomes are evolutionarily conserved

We assembled a high quality draft of the canary genome using a combination of short read and long read sequencing technologies. Since this genome sequence is derived from the DNA of a female canary, the canary genome sequence contains the W chromosome. From comparing the newly sequenced canary genome with the genomes of the zebra finch and other songbirds as well as more distantly related bird genomes we found that, on global scale (synteny, long range collinearity), bird genomes are highly similar. This facilitates the application of collinearity as a bioinformatical tool during the assembly process. Even with distantly related bird species such as zebra finch and chicken, the scaffold building process can be dramatically improved by collinearity. Thus, bird genome assemblies with chromosomal sized superscaffolds are possible without the application of other mapping technologies (Figures 2 and 3). Similar procedures have been practicable in mammalian genomes [80].

There is some disagreement about the number of protein-coding genes between the four published songbird genomes ([41-43] and Refseq NCBI BioProject PRJNA217051 (medium ground finch)) and the canary genome draft. Some of these discrepancies might reflect technical shortcomings of the various sequencing and assembly approaches. Despite this, the alignment of the singing-related transcriptomes (that is, those genes differentially expressed in the HVC or RA in relation to season, testosterone or brain area) onto orthologous transcriptomes shows that all such protein-coding genes have homologous sequences in the zebra finch genome. We did not detect genes resembling vomeronasal receptors, casein milk proteins, salivary-associated proteins or enamel proteins in the canary genome, nor synapsin 1, confirming the results obtained from the zebra finch and chicken genomes [41]. However, we failed to find the duplications (caspase 3, beta secretase, growth hormone) and large expansions of gene families (PAK3, PHF7, PIM1L) coding for brain-expressed proteins that were previously reported for the zebra finch [41,81]. This indicates that a number of these evolutionary novelties appear to be specific to the zebra finch genome sequence, but may not be generalized for other songbird genomes, or might reflect bioinformatics problems (Figure 3). Nevertheless, this global analysis suggests that the evolution of seasonal hormone-sensitive singing and related neuroplasticity of songbirds is only minimally related to the gain and loss of genes, while being highly related to the differential regulation of common genes that exist in all songbird genomes. Interestingly, similar conclusions have been drawn in primates, where the genes of chimpanzees and other primates differ only marginally from those of humans [82], even though only the latter possess speech capabilities.

In contrast to the global similarity of songbird genomes, on the nucleotide resolution level there are considerable species differences, which can impact small sequence motifs like transcription factor binding sites, as shown here for EREs and AREs of canary and zebra finch. Such differences in promoters might lead to major differences in transcriptional regulation, even between closely related species, as discussed below for the control of seasonal singing behaviour of canaries.

### The evolution of area-specific seasonal transcriptomes among song control areas

The canary HVC is responding to the androgenic and estrogenic mode of action of testosterone due to the presence of ERα, estrogen producing aromatase, AR, and androgen producing 5α-reductase (this study and [20,22,66,83]). In contrast, the RA is only androgen sensitive due to the expression of AR (this study and [83]) and 5α-reductase (this study). Furthermore, testosterone up-regulated the expression of the aromatase and of the 5α-reductase gene in HVC.

The seasonality of the RA transcriptome of male canaries mainly concerns intracellular processes of RA cells but not neuronal differentiation. Leaving aside that reports on RA seasonal and testosterone-driven changes in size are conflicting [12,26,84,85], the transcriptome data suggest that such seasonal changes might preliminarily concern non-neuronal compartments of RA and/or neuronal metabolic and transcriptional activity. Although testosterone affects RA morphology, such as the dendritic arborisation of female canaries [47,86], similar effects might be finely graduated in adult males, so that they are not detectable on the level of the entire RA transcriptome.

In contrast to RA, some seasonal HVC gene networks are related to neuronal differentiation and synaptic transmission. Interestingly, most of these neuronal gene networks are testosterone inducible while most of the seasonal biological processes that are not related to neuronal differentiation are not testosterone inducible. Furthermore, the HVC genes that are seasonally down-regulated in response to testosterone are not associated with neuronal differentiation (Additional file 7). Thus, seasonality of gene expression that concerns neuronal differentiation occurs in HVC but not RA and is widely due to testosterone’s activity in HVC. In the white-crowned sparrow, different molecular programs were thought to underlie seasonal neuronal plasticity of HVC and RA [37]. Whether some of the seasonal biological processes of canaries are independent of testosterone’s action but dependent on other seasonal factors, such as long-day photoperiod, acting directly on the HVC, for example, via melatonin [87], remains to be seen.

In particular, the lack of estrogen forming capacity and of estrogen receptors in RA correlates with the lack of seasonal regulation of genes associated with typical neuronal properties (Figure 4E). Thus, we speculate that the combination of ERα and AR in either the same HVC neurons [22] or the same neuronal circuits leads to the seasonal induction of gene networks related to neuronal differentiation and to the seasonal suppression of multiple intracellular processes not directly related to neuronal differentiation during seasons with elevated testosterone. Treatment of adult female canaries with androgens that activate only the AR pathway do not induce song development [88] while testosterone treatment that potentially activates the androgen and estrogen pathway does induce song development in such birds [88,89]. Whether strong transcriptional seasonality in song control areas is linked to the presence of both ER and AR in a song region could be verified in other songbird species that show this pattern. White-crowned sparrows, a seasonal singer of Northern America, evolved the potential to express ERα in HVC [35] and estrogens seem to affect the seasonal-like song pattern via HVC-based activity [90]. Interestingly, similar to the canary, seasonally up-regulated gene networks of the white-crowned sparrow HVC were related, in part, to dendritic arborization, axonogenesis, synaptic organization and electrophysiology [37]. The white-crowned sparrow study is, however, difficult to compare with the present analysis in light of the much higher number of genes that differed significantly between seasons in the canary HVC (Figures 4 and 5; Materials and methods section M11).

Next to the testosterone-driven up-regulation of HVC neuronal differentiation gene networks, seasonal testosterone-dependent down-regulation concerns many more genes than up-regulation and these genes are involved in many different biological processes. Although only speculation, the increased neuronal processes, particularly those involved in the wiring of neuronal networks and in synaptic transmission, and the overall reduced heterogeneity of biological processes of LD males may cause a stereotyped activity of HVC. After all, the main difference between non-breeding and breeding singing is the increased stereotypy of the latter [3,15,16,91] (Figure 1B). Female canaries prefer the fast (testosterone and estrogen-sensitive) stereotyped syllable repetition rates of male canaries [19].

### Gene evolution related to seasonal hormone sensitivity of singing

We used species-specific information from the canary and zebra finch genomes to investigate genomic mechanisms of testosterone-sensitive seasonal singing behaviour by considering the regulatory sites of genes that were seasonally expressed in the canary HVC. In particular, the seasonal testosterone-sensitive HVC-specific gene pool reveals a high percentage of genes, including many genes related to neuronal differentiation and transcription factors, that contain AREs and EREs. Among the seasonal testosterone-induced transcriptomes, genes with EREs are primarily enriched among up-regulated genes while AREs are primarily enriched among down-regulated genes (Figure 5C,D). The frequency of genes with active EREs and AREs might be different from those deduced from the promoter analysis *in silico*. It is likely that the *in silico* approach overestimates the frequency of active hormone responsive sites and therefore underestimates the enrichment of genes with EREs and/or AREs in certain gene pools. Hence, technological advances in the ChIP-seq protocol that enable the detection of such active sites and genes from small samples might help to clarify this problem in the future.

If we calculate the canary specificity of these hormone response elements, about 35% of the ERE- and about 11% of ARE-bearing genes lack these sites in the corresponding zebra finch orthologous promoters (Figure 5F,G; data based on all genes). Thus, the canary-specific evolutionary loss or gain (for example, through point mutations) of EREs and AREs leads to species-specific gene pools that can be regulated by the activation of AR and ERα via testosterone and its androgenic and estrogenic metabolites in HVC. Interestingly, the frequency of such canary-specific AREs among the seasonal HVC genes is lower than that of genes not seasonally regulated in HVC or not expressed in HVC. This suggests that androgen sensitivity of seasonal HVC genes is relatively conserved among canaries and zebra finches and possibly other songbird species. In fact, ARs were found in the HVC of each songbird species studied [92]. Since carduelid (canary) and estrildid (zebra finch) songbird lineages diverged about 40 million years ago [93], a species more closely related to the canary, such as the greenfinch, could be informative regarding the speed of evolution of EREs and AREs.

Because we only quantified potential AR and ERα binding sites based on their nucleotide sequences and within 1 kb of transcription start sites, the difference in the number of genes that have functional hormone binding sites in the canary and zebra finch genome may be larger than what we report here [94-96]. Thus, AR and ERα could regulate transcription factors and, in concert with these or directly, regulate many non-transcription factor genes in the canary HVC due to the evolution of species-specific hormone-responsive *cis*-regulatory sites. The putative androgen- and/or estrogen-sensitive sites of the genome are only partially conserved even between relatively closely related songbird species, similar to the case in mammals [94]. This suggests that conclusions regarding steroid hormone-sensitive gene networks of any particular vertebrate species require genomic information from that species.

Since hormone-responsive genes have, on average, two to four AREs or EREs, the evolution of such sites requires several point mutations or larger genome modifications, such as generation of entire promoters. This suggests a strong driving force behind the type and number of androgen- or estrogen-sensitive genes of canaries. First, our data indicate that direct effects of seasonality that are independent of testosterone production are unlikely to be a driving force (Figure 4F). Likewise, adult song learning in canaries is an unlikely selection pressure for driving these changes, because high levels of testosterone (the condition that leads to the differentially expressed HVC genes) shuts off song learning [97]; the canaries used in these studies learn few new song units in adulthood [16]. Furthermore, due to the expression of most genes expressed in HVC in other brain regions, selection might act on ‘HVC genes’ in relation to hormone-sensitive brain functions executed in other brain regions. In songbirds, one such example might be the estrogen-sensitive auditory processing of the caudal nidopallium [98]. However, since this function also occurs in the zebra finch, it is unlikely to facilitate the evolution of the genomic differences between regulatory sites of genes expressed in the HVC of canaries and zebra finches. A more likely selection force could be female auditory preferences for male song patterns [19], a process that involves the HVC of sexually receptive female canaries [99,100]. However, the observation that HVC transcriptomes of sexually receptive female canaries are strikingly different from those of the males (CF-V and MG, unpublished data) favours the idea that canary-specific hormone sensitivity of genes expressed in the song circuit evolved due to the need for hormone-dependent male song patterning, such as high syllable repetition rates. Genes that are estrogen-sensitive only in canaries and that function in neuron-specific processes (Figure S2A in Additional file 7) are especially good candidates for modifiers of male song patterning which is under sexual selection in canaries.

Our analysis presents the first clear evidence that seasonal reproductive behaviours involve large seasonal hormone-driven differences in gene expression in a defined neural circuit of vertebrates. Further, this transcriptional seasonality differs strongly between regions of a multi-unit neural circuit, which correlates with the regional differences in abundant hormone receptor types. Such evolution of transcriptome development might also explain the large differences in seasonal hormone sensitivity or insensitivity of song features between songbird species [2-11]. Thus, songbird species would be similar to vole species [101] in that there are species differences in brain region-specific expression of hormone receptors that correlate with species differences in behaviour, monogamy-related in voles and singing-related in songbirds [101]. In addition, in songbirds the evolution of species-specific androgen- and estrogen-responsive regulatory sites of genes allows species-specific gene regulation, which is not the case in voles.

## Conclusions

We assembled a high quality 1.2 Gbp draft genome of the canary using a combination of short read and long read sequencing technologies. The use of collinearity as a bioinformatics tool shows that bird genome assemblies with chromosomal sized scaffolds are possible without the application of other mapping technologies. Whole genome alignments between the canary and 13 genomes throughout the Aves class show a much-conserved synteny. However, at the single-base resolution there are considerable species differences which impact small sequence motifs like transcription factor binding sites, as shown here for EREs and AREs, putative binding sites for ERα and AR. The expression of genes with these hormone-response elements is enriched in the seasonal testosterone-induced transcriptome of a major song control region. Since these genes form gene networks related to neuronal differentiation, they are likely to underlie the seasonal testosterone-driven re-differentiation process of the song control region and the song of canaries. Many of these hormone-responsive promoter sites are canary-specific compared with other songbird genomes. The present study demonstrates the need for high-quality genome assemblies to detect gene evolution and evolution of transcriptome development in comparative approaches. It also provides a solid basis for the use of the canary as a model system in studies of molecular neuroscience in general.

## Material and methods

### M1. Animals, testosterone levels, tissue sampling, and RNA isolation

We used zebra finches and common domesticated canaries bred at the Seewiesen animal facility. For the genome sequencing, a female canary was used; for all other procedures, male canaries and zebra finches were used (Additional file 1). Animal handling was carried out in accordance with the European Communities Council Directive 2010/63 EU and the legislation of the state of Upper Bavaria (record number 55.2-1-54-2532-68-12). Reproductively active adult males (one-year-old canaries, n = 21; zebra finches, n =9) were housed pairwise with a female under long-day photoperiod (LD; 14 hours light:10 hours dark). Outside of the breeding season, male canaries (n = 15) were housed pairwise with a female under short-day photoperiod (SD; 10 hours light:14 hours dark). Songs were recorded before killing to confirm that the males had adult typical songs. Six canaries were treated with testosterone. Testicle weight (mean ± standard deviation) was 158 ± 27 mg for the LD canaries, 1.2 ± 0.5 mg for the SD canaries, 0.9 ± 0.6 mg for the SD canaries treated with testosterone, and 58 ± 14 mg for the zebra finches. These testicle weights are typical for reproductively active adult zebra finches and canaries and canaries during the non-breeding season. For testosterone treatment, the birds were subcutaneously implanted with custom-made silastic implants filled with testosterone, as described previously [64].

Testosterone levels (mean ± standard deviation) of the LD males, SD males, and SD + T males were 2.84 ± 1.11 ng/ml plasma, 0.09 ± 0.05 ng/ml plasma, and 6.29 ± 3.79 ng/ml plasma, respectively, on the day of killing. A testosterone radioimmunoassay was carried out as described before for canaries [16].

For RNA-seq, RNA of the HVC, RA, or ENT of three males was pooled for each biological replicate; that is, we used a total of nine male zebra finches and nine male canaries. The other males were used for microarray procedures. The birds were sacrificed quickly with an overdose of isoflurane. Afterwards the brains were quickly removed and snap-frozen over liquid nitrogen. For RNA preparation, brains were cut into four 40 μm para-sagittal sections, mounted each on one slide, followed by two 20 μm sections on a cryostat, mounted on RNase free Superfrost slides as two different series. One series of 20 μm sections was used for Nissl-stainings. These Nissl-stainings were used to verify the location of the HVC, RA, and ENT and to guide the dissection of these areas. The areas were dissected from the 40 μm sections with micro-scalpels (a different one for each brain area to avoid contamination) under a stereo-microscope, transferred into Qiazol (Qiagen Inc., Valencia, CA, USA), and stored at −80°C until RNA preparation.

For *in situ* hybridizations, the left half of each brain of 6 male canaries was cut into 10 adjacent series of 15 μm sections and mounted on RNase free Superfrost slides. One series was used for Nissl-staining; the others were stored at −80°C for *in situ* hybridizations with various probes.

RNA was extracted by the automated Qiacube system using a RNeasy Micro Kit according to the manufacturer’s protocol (Qiagen Inc., Valencia, CA, USA ) using the optional DNA digest step. RNA quality and concentration were assessed using an Agilent Model 2100 Bioanalyzer (Agilent Technologies, Palo Alto, CA, USA) and Nanodrop 1000 spectrometer (Thermo Fisher Scientific, Wilmington, MA, USA), respectively.

### M2. Whole genome shotgun sequencing with Illumina and Roche 454

For whole genome shotgun sequencing, we used the Illumina GAIIx [102] to generate a high sequencing coverage (40×) of the genome based on short reads (2 × 100 bp or 2 × 115 bp) and a lower coverage of the genome (5×) based on long reads (400 bp or 750 bp) from Roche/454 pyro-sequencing technology [103]. All sequencing libraries for whole genome shotgun sequencing were constructed from about 200 μg genomic DNA of a single female domesticated canary. Illumina paired end libraries with 200 to 300 bp insert size as well as the 454 single read libraries were prepared manually according to the manufacturers’ protocols (Illumina TruSeq DNA sample preparation kit, v2, and Roche rapid library prep kit, v2). The Illumina 300 to 500 bp paired-end library was prepared semi-automatically by a Beckman-Coulter SPRI-T robot. Illumina paired end libraries were sequenced on an Illumina GAIIx sequencer.

To reconstruct large genomic scaffolds from the assembled contigs, mate-pair libraries with 2 to 3 kb insert size were prepared manually using Illumina’s biotinylation/circulization protocol (Illumina mate pair v2 library preparation protocol) and sequenced on an Illumina Hiseq2000 sequencer, while Roche/454 20 kb mate pair libs were constructed at Eurofins/MWG using the Cre-recombinase circularization approach from Roche (Roche Diagnostics GmbH, Mannheim, Germany). All 454 libraries were sequenced on GS FLX(+) sequencers.

### M3. Processing of raw sequencing data

While Roche/454 read data can be used for *de novo* assembly directly, Illumina data need to be filtered to improve *de novo* assembly results. Furthermore, filtering Illumina reads is strongly recommended to reduce the need for computing resources (CPU time, RAM and disk space) during genome assembly. During read processing we clipped the reads from 3′ and 5′ ends by choosing the longest region of the read that had no base with quality lower than 11 and trimmed adaptor sequence, if at least 15 bp of the adaptor were found. We used only reads longer than 63 bp (as 64 bp is the minimum read length for assembly with the Celera Assembler; see below). Duplicate fragments generated due to a PCR bias are misleading for genome assemblers and cause false coverage estimates; hence, they were removed and only reads whose first 64 bp had a unique sequence were considered for analysis. Scripts for filtering reads were implemented in Perl and Awk. The average error rate of the trimmed sequence was below 1% according to statistics from Newbler Assembler.

### M4. Genome assembly and scaffolding

We performed different whole genome shotgun assemblies during the different phases of the project using SOAPdenovo (v.1.05) [104], Newbler (v.2.6; Roche) or Celera Assembler (CA6.1) [105]. Among those three assemblers CA6.1 gave the best results in terms of N50 contig and scaffold statistics, while taking the most computing time. The reported canary genome was assembled using CA6.1. The screening of the 2 to 3 kb mate pair data by mapping them to scaffolds of prior assemblies to remove unmated/chimeric read pairs and duplicate read pairs was especially helpful to improve running time and assembly quality of CA6.1.

The genome assembly quality is reflected in the contig N50 length and scaffold N50 length of the canary genome assembly (Table 1). A few potential mis-assemblies in CA6.1 scaffolds became evident as inter-chromosomal rearrangements when mapping them against the zebra finch genome. Scaffolds were split at these locations and subsequently improved by the SSPACE scaffolder [106] with 2 to 3 kb and 20 kb mate pair reads. In a final SSPACE scaffolding step, *de novo* assembled scaffolds were ordered into larger scaffolds, now called superscaffolds, if their ends could be mapped closely (applied windows: 40 ± 20 kb, 200 ± 100 kb, 500 ± 250 kb) and with correct orientation in zebra finch chromosomes. This collinearity (see Materials and methods section M6) to the zebra finch chromosomes resulted in the canary having the qualitatively best bird genome among those produced using second-generation technologies alone (Figures 2 and 3; Table 2; Additional file 2).

### M5. Genome alignment with other bird genomes

The LAST tool [107] was used to align available bird genomes from Ensembl or UCSC databases (budgerigar (*Melopsittacus undulatus*), chicken (*Gallus gallus*), collared flycatcher (*Ficedula albicollis*), medium ground finch (*Geospiza fortis*), turkey (*Meleagris gallopavo*), zebra finch (*Taeniopygia guttata*)) as well as genomes from the CLIMB database (Adelie penguin (*Pygoscelis adeliae*), emperor penguin (*Aptenodytes forsteri*), rock dove (*Columba livia*)) and NCBI GENOME database (mallard (*Anas platyrhynchos*), scarlet macaw (*Ara macao*), Tibetan ground tit (*Pseudopodoces humilis*), white-throated sparrow (*Zonotrichia albicollis*)) to the canary and zebra finch genomes (Figure 3). We found that the speed of whole genome alignments significantly increased when setting the parameter -m from 10 (default) to 1, while maintaining high sensitivity needed to align canary versus chicken and turkey genomes. The raw alignments were clustered according to their reference and query coordinates to remove suboptimal alignments (single_cov2 from TBA/MULTIZ package [108]) and further processed by the BlockDisplaySatsuma script from the Satsuma v.1.17package [109]. Resulting blocks of collinearity were converted to bed format for visualization in the canary genome browser. A complete overview of alignments against the zebra finch chromosomal sequences was done using CIRCOS [46].

### M6. Collinearity improves the genome assembly

While sequence similarity on the nucleotide level goes down to the 70 to 80% range when aligning distantly related bird genomes, collinearity is much more conserved (Figures 2 and 3). Most species have 50% of their genomes in collinear regions larger than 1 Mbp, even when draft assemblies are aligned. To demonstrate that collinearity drastically improves the genome assembly, we used the chicken and zebra finch genome, removing the assembled parts that were based on physical maps or bacterial artificial chromosomes (BACs). We downloaded the zebra finch sequences from the UCSC genome browser. The chromosomal sequences were split into their original scaffolds using information from the assembly track of the browser, and we removed chrUN sequences (it seems that chrUN harbours many sequences redundant with the chromosomal sequences of the assembly). The resulting scaffolds comprised 1,928 scaffolds with a total length of 1,057,275,226 bp. N50 scaffold length was 12.3 Mbp (which is comparable to the canary assembly without collinearity scaffolding). These scaffolds were aligned against the *G. gallus gal4* assembly (UCSC) using the LAST aligner. The alignments were filtered to represent collinear regions of both genomes. We then used several homemade scripts to rebuild the chicken chromosomes with the zebra finch sequence (meaning zebra finch nucleotide sequence ordered according to the chicken genome). The rebuilt genome was split into random paired-end reads of 40 kb (10 × 10^6^ pairs), 200 kb (2 × 10^6^ pairs) and 500 kb (0.8 × 10^6^ pairs) insert size by applying the ‘simulate_reads’ tool from the clc assembly simulator cell v.3.1.0. These reads were used for scaffolding of the original zebra finch scaffolds with the SSPACE v2.0 program (allowing a ±50% change from the original insert size). This resulted in superscaffolds with an N50 length of 36,752,594 bp (a nearly three-fold increase). We compared our collinearity scaffolding results with the zebra finch reference assembly. We found about 93 to 95% (depending on including or excluding unordered pieces of the zebra finch chromosomes in the analysis) of the scaffolds were correctly linked by our approach. The collinearity scaffolding approach was also able to assign 100 zebra finch sequences comprising about 19 Mbp of sequence to scaffolds that were assigned to a chromosome, but not located in the chromosomes of the reference sequence (called chrXY_random in the reference assembly) [41].

### M7. The karyotype of the canary

The karyotype was produced as follows: cell cultures of canaries were performed as described [110] with modifications, using dissociated cells following incubation in collagenase for 1 h. Chromosomes were obtained by standard arrest with colcemid (Gibco, Invitrogen, Carlsbad, CA, USA), hypotonic treatment with 0.075 M KCl, and cell fixation in methanol/acetic acid (3:1). Diploid number definition and karyotype ordering were performed in conventionally stained metaphases (Giemsa 5% in 0.07 M phosphate buffer, pH 6.8).

### M8. Genome annotation

To perform homology/transcript-based annotation we downloaded protein datasets from Uniprot (aves, human), GenBank (aves) and Ensembl (zebra finch, chicken, and turkey) and aligned them with SPALN2.04 [111] to the genome, to the *de novo* assembled transcriptome and to the cufflinks/cuffmerge assembled transcriptome.

Resulting coding sequence (CDS) models from alignments to the genome were clustered and the best scoring model was kept for each cluster. Alignment of proteins against the transcriptome data was used to assign UTR/CDS coordinates to the transcripts’ genomic coordinates and remove putative residual introns as well as remove transcripts that contained stop codons or frame shifts. Assembled transcripts as well as CDS predictions were applied to train the AUGUSTUS gene prediction tool [112], which was used together with hints from transcripts or protein alignments to predict *ab initio* gene models.

To construct a reference gene set, we clustered transcript-based gene models and protein alignment models by their CDS coordinates and selected the best scoring model (the transcript CDS scores were weighted 20% higher to give preference to those models). Finally, AUGUSTUS gene models whose CDS coordinates were non-redundant with transcript-based or homology-based CDS annotations were added to the final gene set.

Automated annotation of the 18,818 reference genes with BLAST2GO [113] resulted in 62% (11,671) BLAST2GO annotated, 9.4% (1,769) annotated but not meeting BLAST2GO criteria for valid annotation, and 16.2% (3,044) having BLAST hits but no GO assignment; 12.4% (2,334) of the genes had no hits to any database. Thus, combining strong and weak assignments, 87.6% (16,484) of the reference genes have an assigned function. To be stringent we based the analysis of the transcriptional activities of the HVC, RA and ENT on a functional (manual) annotation, which identified 10,573 expressed genes for the HVC, 10,572 for the RA, and 11,427 for the ENT. Our annotations increased the number of functionally annotated songbird genes by approximately 40% (4,900 newly annotated genes) compared with previous work [41].

### M9. Repeat analysis and CpG island prediction

To annotate and mask repetitive sequences in the genome, we applied the RepeatModeler package for *de novo* repeat finding [114]. The resulting canary repeat library was combined with known aves repeats from the RepeatMasker libraries and used to mask and annotate repeat elements in the canary genome. CpG island detection was done by CpGplot from the EMBOSS package.

### M10. Transcriptome assembly and differential expression analysis with RNA-seq

We deeply sequenced the transcriptomes of the HVC (n = 3 biological replicates), RA (n = 2 biological replicates), and ENT (n = 3 biological replicates) of canaries and of the HVC (n = 3 biological replicates) of zebra finches using RNA-seq (Additional file 1) and obtained >170 million paired-end reads on average for each sample. All these animals were reproductively active adult males as judged by the size of their testes and their blood testosterone levels (Materials and methods section M1). After quality filtering and adapter removal, we mapped approximately 150 million reads per sample to each reference genome with TopHat using sample-wise insert size parameters, a common splice junction library, and otherwise default parameters. The remaining reads either represented repetitive genomic regions or did not pass the quality threshold. Next, we applied reference-based transcriptome assembly with cufflinks using default parameters [115]. In addition, to include genes and transcripts with very low expression that might otherwise be missed from our transcriptome assemblies, we mapped heterologous protein sequences from public annotations of human, zebra finch and chicken against the canary genome with SPALN [111]. We merged the RNA-seq-derived tissue transcriptomes with cuffmerge and clustered them together with homology predicted transcripts to create organism-specific integrated meta-transcriptomes. The reconstructed transcriptomes are visualized and accessible as combined and tissue-specific tracks in the genome browser [57].

To identify relationships of homologous genes between species, we established two methods. First, we aligned all species meta-transcriptomes against each heterologous species genome and identified co-clustering of potential homologous transcripts within genomic regions with cufflinks. This method turned out to be very robust and, in many cases, the homology could be identified on the level of transcript isoforms. Second, in cases where no homology relationship could be assigned with genomic co-clustering, we used reciprocal blast [116] to identify the likely homologous gene.

To quantify gene expression, we first calculated raw read counts with HTseq and estimated normalized read counts (baseMean) for each of the 12 samples on the basis of the respective organism-specific meta-transcriptomes with DESeq. Differentially expressed genes for each pairwise comparison were identified using the negative-binomial model of read counts as implemented in the DESeq Biocondoctor package. The DESeq R/Bioconductor package [117,118] implements a model based on the negative-binomial distribution and a local regression-based estimation for variance and mean. *P*-values were adjusted for multiple testing with the Benjamini-Hochberg procedure. Differential expression with adjusted *P*-values of <0.05 were considered statistically significant.

### M11. Microarray gene expression and data analysis

Total RNA (100 ng) was processed for hybridization on the microarray using the Ambion WT Expression Kit and the Affymetrix WT Terminal Labeling and Controls Kit. The resulting cDNA was hybridized to the Custom Affymetrix Gene Chip® MPIO-ZF1s520811 Exon Array. For each experimental group and brain area, we made six biological replicates. The 5.7 million zebra finch-specific probes spotted on this array correspond to approximately 232,000 probe sets, hence 33,000 transcripts published on public databases (NCBI-ENSEMBL) and detected in studies performed at the Department of Behavioural Neurobiology, MPIO. The functional final gene annotation was optimized comparing the gene IDs with orthologous human proteins found in EL DORADO homology groups (Genomatix toolbox) and the annotation performed in this study for RNA-seq data. The hybridization was carried out over 16 hours at 45°C and 60 rpm in a GeneChip Hybridization Oven 640. The arrays were washed, stained and scanned using the Affymetrix GeneChip Fluidics Station 450 and Affymetrix GeneChip scanner 3000 7G. The CEL files were generated by the Affymetrix® GeneChip® Command Console® Software (AGCC) and quality control for evaluating the success of individual hybridizations was assessed by the Affymetrix® Expression Console™ software. Affymetrix CEL files were imported into ChipInspector software, version 21 (El Dorado Database version: E26R1204 Genomatix GmbH [119]). Differential expression was analyzed using the group-wise exhaustive analysis with false discovery rate set to zero and 10-significant probe minimum coverage [120].

Since our microarray was designed subsequent to the publication of the zebra finch genome [41], it contains a more complete representation of the zebra finch genes than the microarray used in previous studies (for example, [62]). This and the exon-based design of the presently used microrarray (see above) make comparison with the seasonality study of Stevenson *et al*. [62] difficult.

The microarray data discussed in this publication have been deposited in NCBI’s Gene Expression Omnibus [121] and are accessible through GEO Series accession number GSE50070 [122].

### M12. Validation of area-specific gene expression by *in situ* hybridization

In order to validate the RNA-seq-based expression of genes that were differentially expressed in the HVC, RA and ENT of canary and zebra finch, we designed primers using Primer3web [123]. cDNAs were prepared from total mRNA isolated from a male adult canary forebrain using Superscript III First Strand Synthesis Kit (Invitrogen, Carlsbad*,* CA*,* USA) following the recommended protocol and the amplifications were carried out using Hot FIREPol DNA polymerase (Solis BioDyne, Tartu, Estonia). The amplicons were purified and inserted into the PCRII TOPO vector accurately following the supplied protocol using a TOPO® TA Cloning® Dual Promoter Kit (Invitrogen), host strain TOP10. The inserts were subjected to restriction enzyme analysis and sequencing to confirm the orientation and sequence. The *in situ* hybridization of sagittal cryostat sections was performed as described [20]. For autoradiography, sections were hybridized with the canary- or zebra finch-specific S^35^-labelled RNA anti-sense probes and subsequently exposed to X-ray film (Figure 6). Films were exposed for 4 weeks. For each probe, we used sections of three adult canaries.

In total we performed *in situ* hybridzations for 30 selected genes. For the 60 comparisons (considering the comparison of HVC and ENT and of RA and ENT), the *in situ* hybridizations confirmed the results found in RNA-seq in 53 cases. Here we depict the *in situs* hybridizations of six genes to i) confirm differential gene expression in HVC, RA and ENT derived from the RNA-seq approach, ii) indicate a reason for mismatches, and iii) evaluate the extent of area-specific expression of such genes. The reported genes (Figure 6) are ALDH1A2 (aldehyde dehydrogenase 1 family, member A2; GenBank accession number KF571935), CNTFR (ciliary neurotrophic factor receptor (KF571934)), GLI2 (GLI family zinc finger 2 (KF571936)), GPR83 (G protein-coupled receptor 83 (KF571938)), KCNH5 (potassium voltage-gated channel, subfamily H (eag-related) (KF571937), member 5), and RASGRP1 (RAS guanyl releasing protein 1 (calcium and DAG-regulated) (KF571933)). Expression, either up- or down-regulated in HVC and RA compared with ENT, was as expected from the RNA-seq for all six depicted genes. Nevertheless, down-regulated genes in HVC or RA might still have considerable expression.

### M13. Sample preparations for the creation of the canary brain protein ion library

For the creation of the canary brain protein ion library required for SWATH data processing and protein quantification, tryptic digests were prepared from a pool of RA, HVC-I, HVC-II, ENT, forebrain and cerebellum tissues as described in Shevchenko *et al*. [124]. We pooled the HVC and the entopallium, respectively, of three LD canaries. Briefly, 1 ml of lysis buffer (1% SDS, 10 mM Tris/HCl pH 7.4, 0.15 M NaCl, 1 mM EDTA, 1X Roche Complete Protease Inhibitor in phosphate-buffered saline) was added to the brain tissues and the tissues homogenized for 1 minute on ice using a Dounce glass homogenizer. After 1 h incubation at 4°C, lipids were removed by adding 1.4 ml tri-n-butylphosphate/acetone/methanol mixture (1:12:1) and incubating on ice for 90 minutes. Pellets were successively re-suspended in tri-n-butylphosphate (100%), acetone (100%) and methanol (100%). For trypsin digestion, pellets were re-suspended in 200 μl digestion buffer (1% n-octyl-beta-D-glucopyranoside in 50:50 acetonitrile (ACN)/8 M urea) and proteins were denatured by incubating with 5 mM dithiothreitol (DTT) for 15 minutes at 50°C and alkylated using 10 mM iodoacetamide for 15 minutes at room temperature in the dark. Then, samples were applied to 3 kDa cutoff filters (Millipore, Merck KGaA, Darmstadt, Germany) and impurities removed by repeated centrifugations with 2% ACN in 50 mM ammonium bicarbonate (ABC), 50% ACN in 50 mM ABC and 50 mM ABC. Trypsin was added to the protein samples on the filter (trypsin:protein ratio 1:40) and incubated at 37°C overnight. Tryptic peptides were eluted from the filter by centrifugation and lyophilised. In order to increase the number of peptides identified by liquid chromatography (LC)-tandem MS analysis, a first dimension of separation was achieved by high pH reverse phase chromatography. A reverse phase column (Waters, Milford, MA, USA, BEH C18, 2.1 × 150 mm, 1.7 μm) was utilised in combination with a 10 step gradient (33 minute gradient). Eleven fractions were collected per gradient and supplemented with 1× iRT standard peptide kit (Biognosys, Schlieren, Switzerland) after solvent removal and re-suspension in 5% ACN/0.1% formic acid. A second dimension of separation was achieved by using a nanoLC Reverse Phase column (Eksigent technologies, Dublin, CA, C18 ChromXP nanoLC column 75 μm id × 15 cm, ChromXP C18 3 μm 120 Å) in combination with a 200 minute gradient reaching 35% organic phase in 155 minutes and then 80% organic phase in 5 minutes. Data were acquired on an AB Sciex TripleTOF5600 mass spectrometer in a data dependent acquisition mode (AB Sciex, Framingham, MA, USA). Data processing was performed using ProteinPilot Software 4.5 (AB Sciex) with a search database containing the transcribed canary open reading frames and common contaminant peptides. Peptide identifications with a predicted false discovery rate of <5% were regarded as significant. For the generation of a canary SWATH ion library, the recorded retention time (RT) for each peptide were transformed into indexed RT (iRT) using the iRT-calculator (Biognosys, CH). Then, the eight most intense fragment ions of each peptide were extracted and combined.

### M14. Protein preparation for SWATH-MS analysis and data processing

Protein samples were prepared according to Vowinckel *et al*. [77] from ENT and HVC by incubating the tissues at 90°C for 20 minutes in lysis buffer (0.1 M NaOH, 0.05 M EDTA, 2% SDS, 2% β-mercaptoethanol, 0.1 M acetic acid) and then homogenizing in a TissueLyser (Qiagen) operated at 18 m/s for 2 minutes with 5 mm metal beads (Qiagen). Protein precipitation was performed in 10% trichloroacetic acid and incubation at −80°C for 2 h. Protein pellets were washed twice with 80% acetone before trypsin treatment. Proteins were finally re-suspended in 0.2% Rapigest SF Surfactant (Waters) in 50 mM ABC and quantified using the BCA protein assay kit (Pierce, Rockford, IL, USA). For trypsin digestion 50 μg and 1.6 μg protein were used for ENT and HVC, respectively. Protein samples were denatured by incubating at 60°C for 30 minutes with 5 mM DTT and then alkylated with 10 mM iodoacetamide at room temperature for 30 minutes in the dark. A first aliquot of trypsin was added to the protein samples (enzyme:protein ratio 1:40) and incubated for 2 h. Finally, a second aliquot of trypsin was added to the samples (enzyme:protein ratio 1:40) and digestion performed at 37°C overnight. Surfactant removal was achieved by centrifugation after acidification of the samples with 0.5% trifluoroacetic acid. ACN was added to the samples to a final concentration of 5%.

Targeted proteomics was conducted with minor modifications as described in Gillet *et al*. [76]. Samples were spiked with 1× iRT standard peptide kit and subjected to the nanoLC separation as described above. Data were acquired on an AB Sciex TripleTOF5600 operating in SWATH mode, setting the SWATH m/z acquisition window to 25 Da. Data post-processing was conducted in Skyline [125]. Quantification was carried out by summing the peak areas of the three most intense SWATH transitions per peptide and considering all the peptides identified for each protein of interest. Only unique peptides were considered for quantification of the proteins of interest.

### M15. Gene network analysis

We carried out different analyses using the Genomatix software suite combining several mining sources (MatInspector, Region Miner and Genomatix Pathway Systems (GePS) [126-128]). The canonical pathway and GO-term enrichment analysis were performed using the GePS-Gene Ranker option (Genomatix tool). The full normalized data derived from DESeq analysis for each species (Materials and methods section M10) was filtered by setting the measure of read abundance, that is, expression level cutoff baseMean, at 10 reads, which is a conservative criterion [129]. ClueGO, a Cytoscape plug-in [130-133], was used to interpret the differential gene expression analysis. This application fuses terms of GO as well as KEGG (Kyoto Encyclopedia of Genes and Genomes)/BioCarta pathways and creates a functionally organized GO/pathway term network that can be visualized by functionally grouped terms [63].

### M16. Extracting putative promoter sequences from the genome assemblies for the detection of AREs and EREs

We created subset gtf files for assembled transcripts only present in canary or zebra finch HVC as well as a subset gtf file for HVC genes common to both species. These files were uploaded to the UCSC genome browser (canary and zebra finch). Using the table browser we extracted 1,000 bp of genomic sequence upstream of the putative transcription start sites. Thirty-five percent of the promoter sequences contained gaps in the canary, and 39% contained gaps in the zebra finch. This suggests that both genomes have similar quality regarding promoter regions. For comparisons of ARE and ERE sites between orthologous genes of the canary and zebra finch we considered only those genes without sequence gaps in the promoters of either species.

In order to determine the potential hormone sensitivity of genes, we searched for AREs (V$AREF; matrix names ARE.01, ARE.02, and ARE.03) and EREs (V$EREF; matrix names ER.01, ER.02, and ER.03) within 1,000 bp upstream of the transcription start sites. The matrix selection constraint was the presence of IR3-inverted repeated sequences (palindromes); half-palindromic sites were not considered [34]. Clearly, this approach would discard AR and ER binding sites that are far away from the transcription start sites or that differ from the canonical binding sites. However, ChIP-seq results of the ER-regulated genes in breast cancer showed that most functional ERα binding sites contain EREs [94] and that ERE sites are required for ERα-dependent cell proliferation and differentiation [95]. The putative promoter extracted sequences were analyzed using Genomatix-MatInspector tool [126].

### Major datasets

The following datasets were generated:

1. Canary genome: Frankl-Vilches C, Kuhl H, Werber M, Klages S, Kerick M, Bakker A, de Oliveira EHC, Reusch C, Capuano F, Vowinckel J, Leitner S, Ralser M, Timmermann B, Gahr M, 2013, Genome sequence of the canary (Serinus canaria), PRJEB1766 [134]. In the public domain at the ENA [135].
2. RNA-seq based transcriptomes: Frankl-Vilches C, Kuhl H, Werber M, Klages S, Kerick M, Bakker A, de Oliveira EHC, Reusch C, Capuano F, Vowinckel J, Leitner S, Ralser M, Timmermann B, Gahr M, 2013, Transcriptome sequences of the canary (Serinus canaria), PRJEB4463 [136]. In the public domain at the ENA [135].
3. Microarray based transcriptomes: Frankl-Vilches C, Bakker A, Gahr M, 2013, Expression data from Serinus canaria HVC, RA and entopallium, GSE50070 [122]. In the public domain at the Gene Expression Omnibus [137].

## Additional files

Additional file 1: Table S1. Animals and methods used in the various experiments and procedures. Abbreviations: LD, long-day photoperiod; SD, short-day photoperiod; T, testosterone treatment. M1 to M16 relate to sections in the Materials and methods. N represents the animal numbers. Additional file 2: Table S2. Genome alignments. Statistics of whole genome alignments of various bird species with the canary superscaffolds. A collinear region is a set of alignment blocks with consistent order and orientation along two compared genomes. Values for collinearity regions are strongly influenced by genome assembly quality, that is, two distant but better assembled genomes have higher values than two closely related species with genome assemblies of lower quality. Values for length of alignment blocks are less prone to differences in the quality of genome assemblies. Thus, the species were sorted by the fifth column to better represent their relatedness to the canary genome. ZF (no random) means that we removed canary alignments to unordered pieces of the zebra finch genome, which improved the statistics for this genome. Additional file 3: Table S3. Comparison of N50 length of sequences of the Z chromosomes and of autosomes of the canary and zebra finch. Sequencing a female bird (canary) reduces the sequencing coverage of heterosomes to about 50% of the average genome sequencing coverage and results in lower N50 values for contigs and scaffolds. However, the colinearity superscaffolding that we applied to the canary sequences improved the canary Z scaffolds to sizes that equal the zebra finch Z assembly [41] at the scaffold level. Additional file 4: Figure S1. The karyotype of a female canary consists of 78 autosomes and the Z and W sex chromosome. Note that many chromosomes (19 to 38) are micro-chromosomes. Additional file 5: Table S4. ClueGo analysis of seasonal, testosterone-induced and area-specific transcriptomes of HVC, RA and ENT. The ClueGo visualization assigns the genes of a transcriptome to biological processes. Then biological processes are grouped into functional units named ‘groups’. Each group is named according to the biological process in a group with the highest statistical significance, called Group-GO-Term. Then ClueGo calculates the percentage of a particular group among all groups based on the number of biological processes included in that particular group, termed percentage Group-GO-Term. If several groups are related to the same biological process, these processes are summed for the pie-chart representations depicted in Figure 4 and Additional file 7. Furthermore, groups with ≤1% were summed in one category in the chart representations. The Group-Go-Terms that are related to classical neuronal processes (yellow) are summed as percentage neuronal. *P* < 0.05 for all biological processes. Additional file 6: Table S5. Genes of the testosterone-inducible seasonal up-regulated genes (A, ‘208’ of Figure 5A), seasonal up-regulated genes not testosterone-inducible (B, ‘625’ of Figure 5A), testosterone-inducible seasonal down-regulated genes (C, ‘1,695’ of Figure 5B), and of 1,000 randomly selected genes (D). ‘D’ was used for estimation of the abundance of androgen response elements (AREs) and estrogen response elements (EREs) in the canary and zebra finch genomes. These genes were not found in the HVC transcriptomes. Additional file 7: Figure S2. Gene ontology of HVC transcriptomes that are seasonal testosterone-inducible up-regulated (A, ‘208’ of Figure 5A) or down-regulated (B, ‘1,695’ of Figure 5B). Note that 85% of the biological processes in A are related to processes typical for neuronal plasticity while 0% are related to neuronal differentiation in B. The significant up- or down-regulated transcriptomes were computed as described in section M11 of Materials and methods with stringent statistical settings (false discovery rate = 0) and then analysed with ClueGo (section M11 of Materials and methods) with a significance level set to *P* < 0.05. Biological processes typical of neuronal differentiation and synaptic transmission are depicted in colour; all others are depicted in grayscale. Due to space limitations, we could not include the names of all significant biological processes in the charts but we list them in Additional file 5.

## Abbreviations

ABC: ammonium bicarbonate
ACN: acetonitrile
AR: androgen receptor
ARE: androgen response element
bp: base pair
CA: Celera Assembler
CDS: coding sequence
DTT: dithiothreitol
ENA: European Nucleotide Archive
ENT: entopallium
ERα: estrogen receptor alpha
ERE: estrogen response element
GO: Gene Ontology
iRT: indexed retention time
LC: liquid chromatography
LD: long-day
MS: mass spectrometry
RT: retention time
SD: short-day
T: testosterone
UTR: untranslated region

## References

[1] Aristotle BDMGAPAL (1965). Historia animalium.

[2] Arnold AP (1975). The effects of castration on song development in zebra finches (Poephila guttata). J Exp Zool.

[3] Heid P, Güttinger HR, Pröve E (1985). The influence of castration and testosterone replacement on the song architecture of canaries (Serinus canaria). Z Tierpsychol.

[4] Kunc HP, Foerster K, Vermeirssen ELM, Kempenaers B (2006). Experimentally elevated plasma testosterone levels Do Not influence singing behaviour of male blue tits (Parus caeruleus) during the early breeding season. Ethology.

[5] Maney DL, Lange HS, Raees MQ, Reid AE, Sanford SE (2009). Behavioral phenotypes persist after gonadal steroid manipulation in white-throated sparrows. Horm Behav.

[6] Pinxten R, De Ridder E, Balthazart J, Eens M (2002). Context-dependent effects of castration and testosterone treatment on song in male European starlings. Horm Behav.

[7] Der PE (1974). Einfluß von Kastration und Testosteronsubstitution auf das Sexualverhalten männlicher Zebrafinken (Taeniopygia guttata castanotis Gould). J Ornithol.

[8] Prove E, Immelmann K (1982). Behavioral and hormonal responses of male zebra finches to antiandrogens. Horm Behav.

[9] Strand CR, Ross MS, Weiss SL, Deviche P (2008). Testosterone and social context affect singing behavior but not song control region volumes in adult male songbirds in the fall. Behav Processes.

[10] Van Hout AJ, Pinxten R, Darras VM, Eens M (2012). Testosterone increases repertoire size in an open-ended learner: an experimental study using adult male European starlings (Sturnus vulgaris). Horm Behav.

[11] Walters MJ, Collado D, Harding CF (1991). Oestrogenic modulation of singing in male zebra finches: differential effects on directed and undirected songs. Anim Behav.

[12] Leitner S, Voigt C, Garcia-Segura LM, Van’t Hof T, Gahr M (2001). Seasonal activation and inactivation of song motor memories in wild canaries is not reflected in neuroanatomical changes of forebrain song areas. Horm Behav.

[13] Voigt C, Leitner S, Gahr M (2006). Repertoire and structure of duet and solo songs in cooperatively breeding white-browed sparrow weavers. Behaviour.

[14] Gahr M (2014). How hormone-sensitive are bird songs and what are the underlying mechanisms?. Acta Acustica united with Acustica.

[15] Nottebohm F, Nottebohm ME, Crane LA, Wingfield JC (1987). Seasonal changes in gonadal hormone levels of adult male canaries and their relation to song. Behav Neural Biol.

[16] Voigt C, Leitner S (2008). Seasonality in song behaviour revisited: Seasonal and annual variants and invariants in the song of the domesticated canary (Serinus canaria). Horm Behav.

[17] Güttinger HR (1985). Consequences of domestication on the song structures in the canary. Behaviour.

[18] Fusani L, Metzdorf R, Hutchison JB, Gahr M (2003). Aromatase inhibition affects testosterone-induced masculinization of song and the neural song system in female canaries. J Neurobiol.

[19] Vallet E, Kreutzer M (1995). Female canaries are sexually responsive to special song phrases. Anim Behav.

[20] Gahr M, Metzdorf R (1997). Distribution and dynamics in the expression of androgen and estrogen receptors in vocal control systems of songbirds. Brain Res Bull.

[21] Gahr M, Flugge G, Guttinger HR (1987). Immunocytochemical localization of estrogen-binding neurons in the songbird brain. Brain Res.

[22] Gahr M (1990). Localization of androgen receptors and estrogen receptors in the same cells of the songbird brain. Proc Natl Acad Sci U S A.

[23] Ball GF, Bernard DJ, Foidart A, Lakaye B, Balthazart J (1999). Steroid sensitive sites in the avian brain: does the distribution of the estrogen receptor alpha and beta types provide insight into their function?. Brain Behav Evol.

[24] Nottebohm F, Stokes TM, Leonard CM (1976). Central control of song in the canary. Serinus canarius. J Comp Neurol.

[25] Wild JM (2004). Functional neuroanatomy of the sensorimotor control of singing. Ann N Y Acad Sci.

[26] Gahr M (1990). Delineation of a brain nucleus: comparisons of cytochemical, hodological, and cytoarchitectural views of the song control nucleus HVc of the adult canary. J Comp Neurol.

[27] Nottebohm F (1981). A brain for all seasons: cyclical anatomical changes in song control nuclei of the canary brain. Science.

[28] Kirn JR, Alvarez-Buylla A, Nottebohm F (1991). Production and survival of projection neurons in a forebrain vocal center of adult male canaries. J Neurosci.

[29] Sartor JJ, Ball GF (2005). Social suppression of song is associated with a reduction in volume of a song-control nucleus in European starlings (Sturnus vulgaris). Behav Neurosci.

[30] Goldman SA, Nottebohm F (1983). Neuronal production, migration, and differentiation in a vocal control nucleus in the adult female canary brain. Proc Natl Acad Sci U S A.

[31] Gnerre S, Maccallum I, Przybylski D, Ribeiro FJ, Burton JN, Walker BJ (2011). High-quality draft assemblies of mammalian genomes from massively parallel sequence data. Proc Natl Acad Sci U S A.

[32] McEwen BS, Davis PG, Parsons B, Pfaff DW (1979). The brain as a target for steroid hormone action. Annu Rev Neurosci.

[33] Arnold AP, Nottebohm F, Pfaff DW (1976). Hormone concentrating cells in vocal control and other areas of the brain of the zebra finch (Poephila guttata). J Comp Neurol.

[34] Mangelsdorf DJ, Thummel C, Beato M, Herrlich P, Schutz G, Umesono K (1995). The nuclear receptor superfamily: the second decade. Cell.

[35] Gahr M, Guttinger HR, Kroodsma DE (1993). Estrogen receptors in the avian brain: survey reveals general distribution and forebrain areas unique to songbirds. J Comp Neurol.

[36] Drnevich J, Replogle KL, Lovell P, Hahn TP, Johnson F, Mast TG (2012). Impact of experience-dependent and -independent factors on gene expression in songbird brain. Proc Natl Acad Sci U S A.

[37] Thompson CK, Meitzen J, Replogle K, Drnevich J, Lent KL, Wissman AM (2012). Seasonal changes in patterns of gene expression in avian song control brain regions. PLoS One.

[38] International Chicken Genome Sequencing C (2004). Sequence and comparative analysis of the chicken genome provide unique perspectives on vertebrate evolution. Nature.

[39] Dalloul RA, Long JA, Zimin AV, Aslam L, Beal K, Le Blomberg A (2010). Multi-platform next-generation sequencing of the domestic turkey (Meleagris gallopavo): genome assembly and analysis. PLoS Biol.

[40] Huang Y, Li Y, Burt DW, Chen H, Zhang Y, Qian W (2013). The duck genome and transcriptome provide insight into an avian influenza virus reservoir species. Nat Genet.

[41] Warren WC, Clayton DF, Ellegren H, Arnold AP, Hillier LW, Kunstner A (2010). The genome of a songbird. Nature.

[42] Qu Y, Zhao H, Han N, Zhou G, Song G, Gao B (2013). Ground tit genome reveals avian adaptation to living at high altitudes in the Tibetan plateau. Nat Commun.

[43] Poelstra JW, Vijay N, Bossu CM, Lantz H, Ryll B, Muller I (2014). The genomic landscape underlying phenotypic integrity in the face of gene flow in crows. Science.

[44] Suh A, Paus M, Kiefmann M, Churakov G, Franke FA, Brosius J (2011). Mesozoic retroposons reveal parrots as the closest living relatives of passerine birds. Nat Commun.

[45] Zhang G, Parker P, Li B, Li H, Wang J. The genome of Darwin’s Finch (Geospiza fortis). GigaScience. 2012. http://dx.doi.org/10.5524/100040.

[46] Romanov MN, Dodgson JB, Gonser RA, Tuttle EM (2011). Comparative BAC-based mapping in the white-throated sparrow, a novel behavioral genomics model, using interspecies overgo hybridization. BMC Res Notes.

[47] Hackett SJ, Kimball RT, Reddy S, Bowie RC, Braun EL, Braun MJ (2008). A phylogenomic study of birds reveals their evolutionary history. Science.

[48] 48.Genome sequence of the canary (Serinus canaria) http://www.ebi.ac.uk/ena/data/search?query=GCA_000534875.

[49] Ayers KL, Davidson NM, Demiyah D, Roeszler KN, Grutzner F, Sinclair AH (2013). RNA sequencing reveals sexually dimorphic gene expression before gonadal differentiation in chicken and allows comprehensive annotation of the W-chromosome. Genome Biol.

[50] Auer H, Mayr B, Lambrou M, Schleger W (1987). An extended chicken karyotype, including the NOR chromosome. Cytogenet Cell Genet.

[51] Fritschi S, Stranzinger G (1985). Fluorescent chromosome banding in inbred chicken: quinacrine bands, sequential chromomycin and Dapi bands. Theor Appl Genet.

[52] Kaelbling M, Fechheimer NS (1983). Synaptonemal complexes and the chromosome complement of domestic fowl, Gallus domesticus. Cytogenet Cell Genet.

[53] de Leon FA P, Li Y, Weng Z (1992). Early and late replicative chromosomal banding patterns of Gallus domesticus. J Hered.

[54] Ohno S, Stenius C, Christian LC, Becak W, Becak ML (1964). Chromosomal uniformity in the avian subclass carinatae. Chromosoma.

[55] Itoh Y, Arnold AP (2005). Chromosomal polymorphism and comparative painting analysis in the zebra finch. Chromosome Res.

[56] Kent WJ, Sugnet CW, Furey TS, Roskin KM, Pringle TH, Zahler AM (2002). The human genome browser at UCSC. Genome Res.

[57] Canary Genome Browser. http://public-genomes-ngs.molgen.mpg.de.

[58] Hilliard Austin T, Miller Julie E, Fraley ER, Horvath S, White SA (2012). Molecular microcircuitry underlies functional specification in a basal ganglia circuit dedicated to vocal learning. Neuron.

[59] Kato M, Okanoya K (2010). Molecular characterization of the song control nucleus HVC in Bengalese finch brain. Brain Res.

[60] Li X, Wang XJ, Tannenhauser J, Podell S, Mukherjee P, Hertel M (2007). Genomic resources for songbird research and their use in characterizing gene expression during brain development. Proc Natl Acad Sci U S A.

[61] Lovell PV, Clayton DF, Replogle KL, Mello CV (2008). Birdsong “transcriptomics”: neurochemical specializations of the oscine song system. PLoS One.

[62] Stevenson TJ, Replogle K, Drnevich J, Clayton DF, Ball GF (2012). High throughput analysis reveals dissociable gene expression profiles in two independent neural systems involved in the regulation of social behavior. BMC Neurosci.

[63] Bindea G, Mlecnik B, Hackl H, Charoentong P, Tosolini M, Kirilovsky A (2009). ClueGO: a Cytoscape plug-in to decipher functionally grouped gene ontology and pathway annotation networks. Bioinformatics.

[64] Hartog TE, Dittrich F, Pieneman AW, Jansen RF, Frankl-Vilches C, Lessmann V (2009). Brain-derived neurotrophic factor signaling in the HVC is required for testosterone-induced song of female canaries. J Neurosci.

[65] Balthazart J, Gahr M, Surlemont C (1989). Distribution of estrogen receptors in the brain of the Japanese quail: an immunocytochemical study. Brain Res.

[66] Fusani L, Van’t Hof T, Hutchison JB, Gahr M (2000). Seasonal expression of androgen receptors, estrogen receptors, and aromatase in the canary brain in relation to circulating androgens and estrogens. J Neurobiol.

[67] Zhang C, Gao J, Zhang H, Sun L, Peng G (2012). Robo2-slit and Dcc-netrin1 coordinate neuron axonal pathfinding within the embryonic axon tracts. J Neurosci.

[68] Byun J, Kim BT, Kim YT, Jiao Z, Hur EM, Zhou FQ (2012). Slit2 inactivates GSK3beta to signal neurite outgrowth inhibition. PLoS One.

[69] Ren D (2011). Sodium leak channels in neuronal excitability and rhythmic behaviors. Neuron.

[70] Chen L, Fu Y, Ren M, Xiao B, Rubin CS (2011). A RasGRP, C. elegans RGEF-1b, couples external stimuli to behavior by activating LET-60 (Ras) in sensory neurons. Neuron.

[71] Kressler D, Schreiber SN, Knutti D, Kralli A (2002). The PGC-1-related protein PERC is a selective coactivator of estrogen receptor alpha. J Biol Chem.

[72] Li X, Zhu C, Tu WH, Yang N, Qin H, Sun Z (2011). ZMIZ1 preferably enhances the transcriptional activity of androgen receptor with short polyglutamine tract. PLoS One.

[73] Teramitsu I, Kudo LC, London SE, Geschwind DH, White SA (2004). Parallel FoxP1 and FoxP2 expression in songbird and human brain predicts functional interaction. J Neurosci.

[74] Chen Q, Heston JB, Burkett ZD, White SA (2013). Expression analysis of the speech-related genes FoxP1 and FoxP2 and their relation to singing behavior in two songbird species. J Exp Biol.

[75] Schwanhausser B, Busse D, Li N, Dittmar G, Schuchhardt J, Wolf J (2011). Global quantification of mammalian gene expression control. Nature.

[76] Gillet LC, Navarro P, Tate S, Rost H, Selevsek N, Reiter L (2012). Targeted data extraction of the MS/MS spectra generated by data-independent acquisition: a new concept for consistent and accurate proteome analysis. Mol Cell Proteomics.

[77] Vowinckel J, Capuano F, Campbell K, Deery MJ, Lilley KS, Ralser M (2013). The beauty of being (label)-free: sample preparation methods for SWATH-MS and next-generation targeted proteomics. F1000 Res.

[78] Kushner PJ, Agard DA, Greene GL, Scanlan TS, Shiau AK, Uht RM (2000). Estrogen receptor pathways to AP-1. J Steroid Biochem Mol Biol.

[79] Porter W, Saville B, Hoivik D, Safe S (1997). Functional synergy between the transcription factor Sp1 and the estrogen receptor. Mol Endocrinol.

[80] Kim J, Larkin DM, Cai AQ, Zhang Y, Ge RL (2013). Reference-assisted chromosome assembly. Proc Natl Acad Sci U S A.

[81] Kong L, Lovell PV, Heger A, Mello CV, Ponting CP (2010). Accelerated evolution of PAK3- and PIM1-like kinase gene families in the zebra finch. Taeniopygia guttata. Mol Biol Evol.

[82] Prufer K, Munch K, Hellmann I, Akagi K, Miller JR, Walenz B (2012). The bonobo genome compared with the chimpanzee and human genomes. Nature.

[83] Balthazart J, Foidart A, Wilson EM, Ball GF (1992). Immunocytochemical localization of androgen receptors in the male songbird and quail brain. J Comp Neurol.

[84] Nottebohm F (1980). Testosterone triggers growth of brain vocal control nuclei in adult female canaries. Brain Res.

[85] Sartor JJ, Balthazart J, Ball GF (2005). Coordinated and dissociated effects of testosterone on singing behavior and song control nuclei in canaries (Serinus canaria). Horm Behav.

[86] DeVoogd T, Nottebohm F (1981). Gonadal hormones induce dendritic growth in the adult avian brain. Science.

[87] Jansen R, Metzdorf R, van der Roest M, Fusani L, ter Maat A, Gahr M (2005). Melatonin affects the temporal organization of the song of the zebra finch. FASEB J.

[88] DeVoogd TJ (1991). Endocrine mdulation of the development and adult function of the avian song system. Psychoneuroendocrinol.

[89] Fusani L, Gahr M (2006). Hormonal influence on song structure and organization: the role of estrogen. Neuroscience.

[90] Meitzen J, Moore IT, Lent K, Brenowitz EA, Perkel DJ (2007). Steroid hormones act transsynaptically within the forebrain to regulate neuronal phenotype and song stereotypy. J Neurosci.

[91] Fusani L, Van’t Hof T, Hutchison JB (2003). Season-related changes in circulating androgen, brain aromatase, and perch-calling in male ring doves. Gen Comp Endocrinol.

[92] Metzdorf R, Gahr M, Fusani L (1999). Distribution of aromatase, estrogen receptor, and androgen receptor mRNA in the forebrain of songbirds and nonsongbirds. J Comp Neurol.

[93] Vellema M, Hertel M, Urbanus SL, Van der Linden A, Gahr M (2014). Evaluating the predictive value of doublecortin as a marker for adult neurogenesis in canaries (Serinus canaria). J Comp Neurol.

[94] Lin Z, Reierstad S, Huang CC, Bulun SE (2007). Novel estrogen receptor-alpha binding sites and estradiol target genes identified by chromatin immunoprecipitation cloning in breast cancer. Cancer Res.

[95] Nott SL, Huang Y, Li X, Fluharty BR, Qiu X, Welshons WV (2009). Genomic responses from the estrogen-responsive element-dependent signaling pathway mediated by estrogen receptor alpha are required to elicit cellular alterations. J Biol Chem.

[96] Stender JD, Kim K, Charn TH, Komm B, Chang KC, Kraus WL (2010). Genome-wide analysis of estrogen receptor alpha DNA binding and tethering mechanisms identifies Runx1 as a novel tethering factor in receptor-mediated transcriptional activation. Mol Cell Biol.

[97] Güttinger H, Fuchs H, Schwager G (1990). Das Gesangslernen und seine Beziehung zur Gehirnentwicklung beim Kanarienvogel (Serinus canaria). Die Vogelwarte.

[98] Vellema M, Ko MC, Frankl-Vilches C, Gahr M (2014). What makes a marker a good marker? Commentary on Balthazart J and Ball G (2014): Doublecortin is a highly valuable endogenous marker of adult neurogenesis in canaries. Brain Behav Evol 84:1–4. Brain Behav Evol.

[99] Brenowitz EA (1991). Altered perception of species-specific song by female birds after lesions of a forebrain nucleus. Science.

[100] Del Negro C, Kreutzer M, Gahr M (2000). Sexually stimulating signals of canary (Serinus canaria) songs: evidence for a female-specific auditory representation in the HVc nucleus during the breeding season. Behav Neurosci.

[101] Young LJ, Wang Z (2004). The neurobiology of pair bonding. Nat Neurosci.

[102] Bentley DR, Balasubramanian S, Swerdlow HP, Smith GP, Milton J, Brown CG (2008). Accurate whole human genome sequencing using reversible terminator chemistry. Nature.

[103] Margulies M, Egholm M, Altman WE, Attiya S, Bader JS, Bemben LA (2005). Genome sequencing in microfabricated high-density picolitre reactors. Nature.

[104] Li R, Zhu H, Ruan J, Qian W, Fang X, Shi Z (2010). De novo assembly of human genomes with massively parallel short read sequencing. Genome Res.

[105] Miller JR, Delcher AL, Koren S, Venter E, Walenz BP, Brownley A (2008). Aggressive assembly of pyrosequencing reads with mates. Bioinformatics.

[106] Boetzer M, Henkel CV, Jansen HJ, Butler D, Pirovano W (2011). Scaffolding pre-assembled contigs using SSPACE. Bioinformatics.

[107] Kiełbasa SM, Wan R, Sato K, Horton P, Frith MC (2011). Adaptive seeds tame genomic sequence comparison. Genome Res.

[108] Reiner A, Perkel DJ, Mello CV, Jarvis ED (2004). Songbirds and the revised avian brain nomenclature. Ann N Y Acad Sci.

[109] Grabherr MG, Russell P, Meyer M, Mauceli E, Alfoldi J, Di Palma F (2010). Genome-wide synteny through highly sensitive sequence alignment: Satsuma. Bioinformatics.

[110] Saski M, Ikechi T, Makino S (1968). A feather pulp culture technique for avian chromosomes, with notes on the chromosomes of the peafowl and the ostrich. Experientia.

[111] Gotoh O (2008). A space-efficient and accurate method for mapping and aligning cDNA sequences onto genomic sequence. Nucleic Acids Res.

[112] Stanke M, Diekhans M, Baertsch R, Haussler D (2008). Using native and syntenically mapped cDNA alignments to improve de novo gene finding. Bioinformatics.

[113] Conesa A, Gotz S, Garcia-Gomez JM, Terol J, Talon M, Robles M (2005). Blast2GO: a universal tool for annotation, visualization and analysis in functional genomics research. Bioinformatics.

[114] RepeatMasker. http://www.repeatmasker.org.

[115] Trapnell C, Roberts A, Goff L, Pertea G, Kim D, Kelley DR (2012). Differential gene and transcript expression analysis of RNA-seq experiments with TopHat and Cufflinks. Nat Protoc.

[116] Altschul SF, Gish W, Miller W, Myers EW, Lipman DJ (1990). Basic local alignment search tool. J Mol Biol.

[117] Anders S, Huber W (2010). Differential expression analysis for sequence count data. Genome Biol.

[118] Anders S, Reyes A, Huber W (2012). Detecting differential usage of exons from RNA-seq data. Genome Res.

[119] genomatix. http://www.genomatix.de.

[120] Cohen CD, Lindenmeyer MT, Eichinger F, Hahn A, Seifert M, Moll AG (2008). Improved elucidation of biological processes linked to diabetic nephropathy by single probe-based microarray data analysis. PLoS One.

[121] Edgar R, Domrachev M, Lash AE (2002). Gene Expression Omnibus: NCBI gene expression and hybridization array data repository. Nucleic Acids Res.

[122] Expression data from Serinus canaria HVC, RA and Entopallium http://www.ncbi.nlm.nih.gov/geo/query/acc.cgi?acc=GSE50070.

[123] Primer3web. http://primer3.wi.mit.edu.

[124] Shevchenko G, Musunuri S, Wetterhall M, Bergquist J (2012). Comparison of extraction methods for the comprehensive analysis of mouse brain proteome using shotgun-based mass spectrometry. J Proteome Res.

[125] MacLean B, Tomazela DM, Shulman N, Chambers M, Finney GL, Frewen B (2010). Skyline: an open source document editor for creating and analyzing targeted proteomics experiments. Bioinformatics.

[126] Cartharius K, Frech K, Grote K, Klocke B, Haltmeier M, Klingenhoff A (2005). MatInspector and beyond: promoter analysis based on transcription factor binding sites. Bioinformatics.

[127] Michelhaugh SK, Lipovich L, Blythe J, Jia H, Kapatos G, Bannon MJ (2011). Mining Affymetrix microarray data for long non-coding RNAs: altered expression in the nucleus accumbens of heroin abusers. J Neurochem.

[128] Sharad S, Srivastava A, Ravulapalli S, Parker P, Chen Y, Li H (2011). Prostate cancer gene expression signature of patients with high body mass index. Prostate Cancer Prostatic Dis.

[129] Voigt C, Gahr M, Leitner S, Lutermann H, Bennett N (2014). Breeding status and social environment differentially affect the expression of sex steroid receptor and aromatase mRNA in the brain of female Damaraland mole-rats. Front Zool.

[130] Cline MS, Smoot M, Cerami E, Kuchinsky A, Landys N, Workman C (2007). Integration of biological networks and gene expression data using Cytoscape. Nat Protoc.

[131] Saito R, Smoot ME, Ono K, Ruscheinski J, Wang PL, Lotia S (2012). A travel guide to Cytoscape plugins. Nat Methods.

[132] Shannon P, Markiel A, Ozier O, Baliga NS, Wang JT, Ramage D (2003). Cytoscape: a software environment for integrated models of biomolecular interaction networks. Genome Res.

[133] Smoot ME, Ono K, Ruscheinski J, Wang PL (2011). Ideker. Cytoscape 2.8: new features for data integration and network visualization. Bioinformatics.

[134] ENA: Genome sequence of the canary (Serinus canaria). http://www.ebi.ac.uk/ena/data/view/PRJEB1766

[135] European Nucleotide Archive. http://www.ebi.ac.uk/ena/.

[136] Transcriptome sequencing of Serinus Canaria and Taeniopygia guttata brain tissues. http://www.ebi.ac.uk/ena/data/view/PRJEB4463

[137] Gene expression Omnibus. http://www.ncbi.nlm.nih.gov/geo/.

