# Supplementary material for: Using the canary genome to decipher the evolution of hormone-sensitive gene regulation in seasonal singing birds
Source: Genome Biol. 2015 Jan 29;16(1):19. doi: 10.1186/s13059-014-0578-9 (PMC4373106; doi:10.1186/s13059-014-0578-9)
Supplement: Additional file 1: Table S1. — Animals and methods used in the various experiments and procedures. Abbreviations: LD, long-day photoperiod; SD, short-day photoperiod; T, testosterone treatment. M1 to M16 relate to sections in the Materials and methods. N represents the animal numbers. [file 13059_2014_578_MOESM1_ESM.pdf]

Analysis	Methods	Canary female	Canary male				Zebra finch male		Major findings
		N	N	LD samples	SD samples	SD+T samples	N	samples	
Genome	M1, M2-M9	1							1.2 Gbp genome; 1.69 Mbp W chromosome; 18,818 protein coding genes
RNAseq HVC	M10, M15, M16		9	3x3*			9	3x3*	Species-specific AREs and EREs. Enrichment of genes with ARE and ERE in testosterone-sensitive HVC transcriptomes
RNAseq RA	M10, M15, M16			2x3*				2x3*	
RNAseq ENT	M10, M15, M16			3x3*				3x3*	
RNA-microarray HVC	M11, M15		18	6	6	6			HVC-differential seasonal testosterone-induced genes are related to neural and neuronal plasticity. Validation of the RNAseq expression data.
RNA-microarray RA				6	6	6			
RNA-microarray ENT	M11, M15			6	6	6			
In-situ hybridizations	M12		6	3	3				Validation of area-specific gene expression
Protein HVC	M13-M14		3	1x3*					Correlation between gene expression and protein levels of HVC and RA
Protein ENT	M13-M14			1x3*					

* = 3 biological samples were pooled before analysis
