# Supplementary material for: Using the canary genome to decipher the evolution of hormone-sensitive gene regulation in seasonal singing birds
Source: Genome Biol. 2015 Jan 29;16(1):19. doi: 10.1186/s13059-014-0578-9 (PMC4373106; doi:10.1186/s13059-014-0578-9)
Supplement: Additional file 2: Table S2. — Genome alignments. Statistics of whole genome alignments of various bird species with the canary superscaffolds. A collinear region is a set of alignment blocks with consistent order and orientation along two compared genomes. Values for collinearity regions are strongly influenced by genome assembly quality, that is, two distant but better assembled genomes have higher values than two closely related species with genome assemblies of lower quality. Values for length of alignment blocks are less prone to differences in the quality of genome assemblies. Thus, the species were sorted by the fifth column to better represent their relatedness to the canary genome. ZF (no random) means that we removed canary alignments to unordered pieces of the zebra finch genome, which improved the statistics for this genome. [file 13059_2014_578_MOESM2_ESM.pdf]

aligned species	CAN sequence covered by collinear regions	largest collinear region	N50 collinear region length	CAN sequence covered by alignment blocks	largest alignment block	N50 alignment block length	average % nucleotide identity of alignment
**Ground finch**	1,007,261,357 (89.03%)	17,993,560	2,357,661	828,553,970 (73.23%)	51,229	4,933	90.47%
**ZF (no random)**	975,908,470 (86.26%)	20,770,821	2,198,027	785,773,747 (69,45%)	40,686	3,725	87.97%
**Adelie penguin**	995,705,335 (88.00%)	7,648,194	1,330,176	785,296,928 (69.41%)	19,470	1,428	78.67%
**Emperor penguin**	1,002,184,706 (88.58%)	10,697,713	1,372,433	783,035,446 (69.21%)	23,230	1,429	78.85%
**Zebra finch (ZF)**	993,034,350 (87.78%)	9,912,053	1,231,989	776,753,394 (68.66%)	41,519	3,730	87.95%
**Rock dove**	998,796,821 (88.28%)	6,988,323	1,087,578	681,040,241 (60.19%)	19,278	1,095	75.76%
**Budgerigar**	985,728,753 (87.13%)	9,998,822	1,339,601	648,921,248 (57.36%)	20,965	1,099	75.17%
**Mallard**	983,346,013 (86.92%)	3,683,034	742,967	485,488,945 (42.91%)	20,971	839	73.92%
**Chicken**	988,238,072 (87.35%)	14,933,872	2,241,173	388,443,473 (34.33%)	18,437	773	73.50%
**Turkey**	956,395,519 (84.54%)	3,653,276	599,138	353,305,655 (31.23%)	21,111	740	73.56%

Supplementary Tab. 2 - Frankl et al
