# Supplementary figures and images for: Using the canary genome to decipher the evolution of hormone-sensitive gene regulation in seasonal singing birds
Source: Genome Biol. 2015 Jan 29;16(1):19. doi: 10.1186/s13059-014-0578-9 (PMC4373106; doi:10.1186/s13059-014-0578-9)

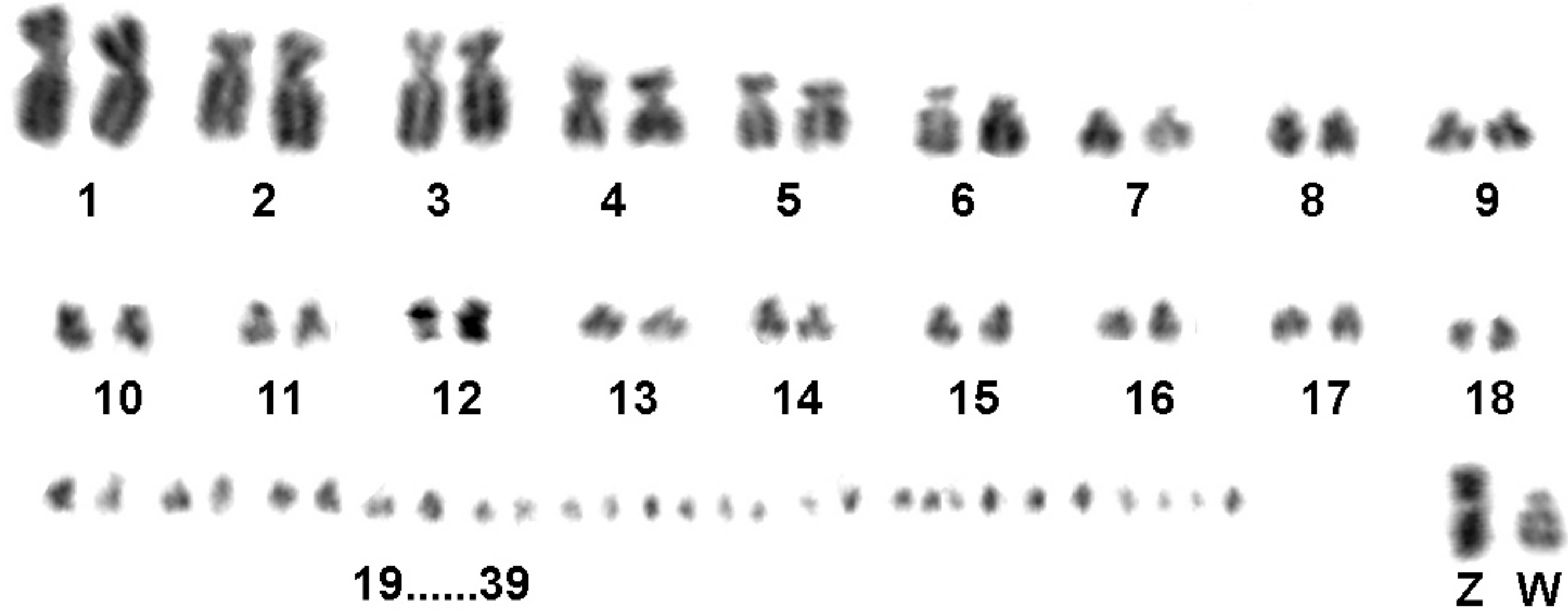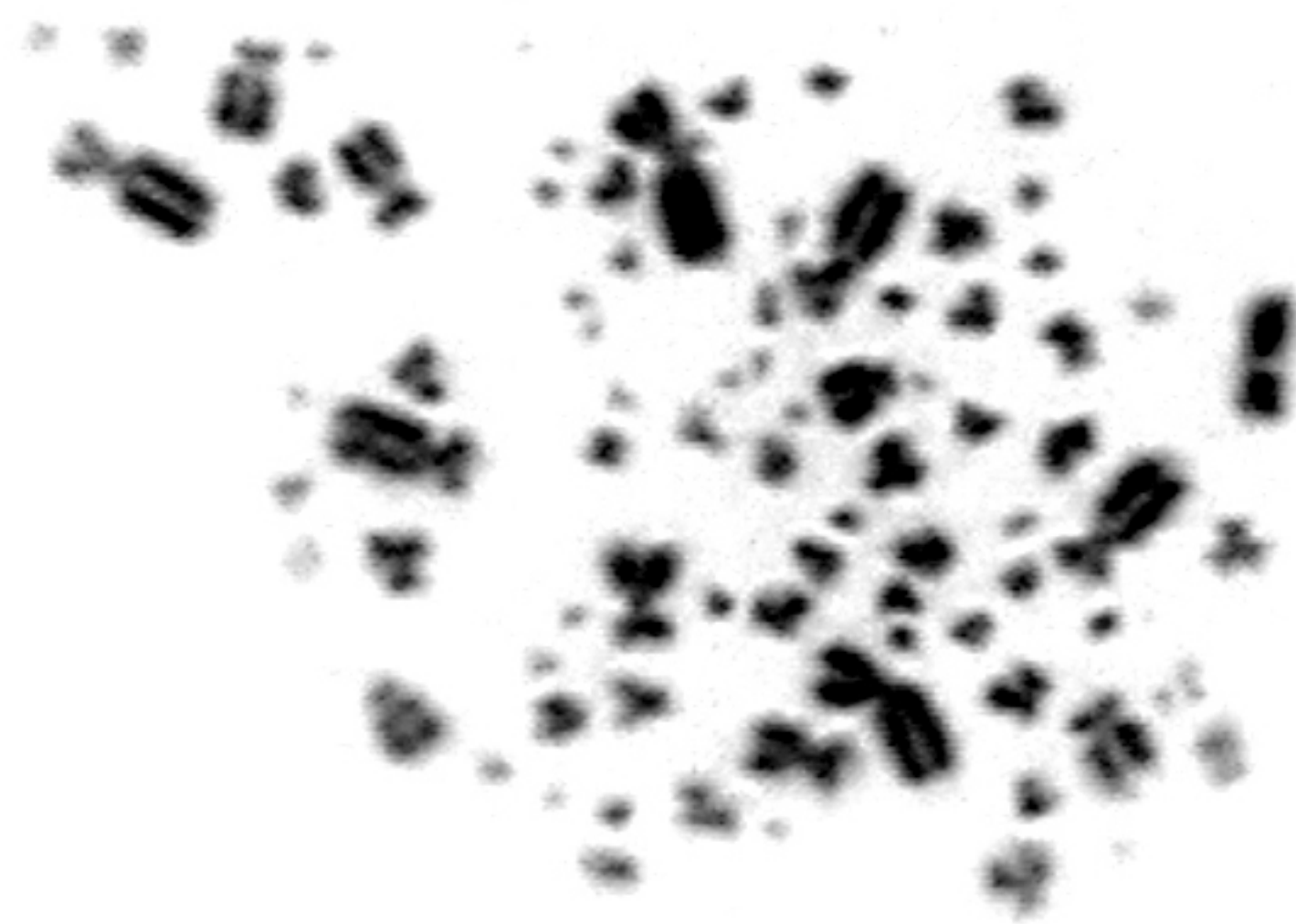

Supplement: Additional file 4: Figure S1. — The karyotype of a female canary consists of 78 autosomes and the Z and W sex chromosome. Note that many chromosomes (19 to 38) are micro-chromosomes. [file 13059_2014_578_MOESM4_ESM.pdf]
