# Supplementary material for: Using the canary genome to decipher the evolution of hormone-sensitive gene regulation in seasonal singing birds
Source: Genome Biol. 2015 Jan 29;16(1):19. doi: 10.1186/s13059-014-0578-9 (PMC4373106; doi:10.1186/s13059-014-0578-9)
Supplement: Additional file 7: Figure S2. — Gene ontology of HVC transcriptomes that are seasonal testosterone-inducible up-regulated (A, ‘208’ of Figure 5A) or down-regulated (B, ‘1,695’ of Figure 5B). Note that 85% of the biological processes in A are related to processes typical for neuronal plasticity while 0% are related to neuronal differentiation in B. The significant up- or down-regulated transcriptomes were computed as described in section M11 of Materials and methods with stringent statistical settings (false discovery rate = 0) and then analysed with ClueGo (section M11 of Materials and methods) with a significance level set to P < 0.05. Biological processes typical of neuronal differentiation and synaptic transmission are depicted in colour; all others are depicted in grayscale. Due to space limitations, we could not include the names of all significant biological processes in the charts but we list them in Additional file 5. [file 13059_2014_578_MOESM7_ESM.pdf]

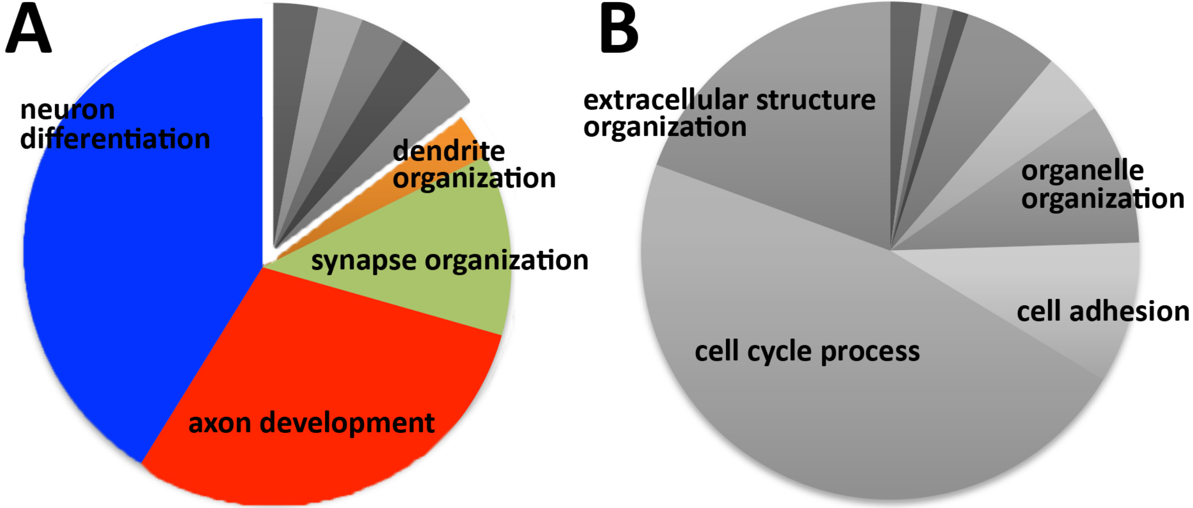

Supp. Fig. 2 - Frankl et al
